# Supplementary material for: Mixed methods evaluation of the Getting it Right First Time programme in elective orthopaedic surgery in England: an analysis from the National Joint Registry and Hospital Episode Statistics
Source: BMJ Open. 2022 Jun 16;12(6):e058316. doi: 10.1136/bmjopen-2021-058316 (PMC9207914; doi:10.1136/bmjopen-2021-058316)
Supplement: Supplementary data [file bmjopen-2021-058316supp001.pdf]

Appendix 1: Case study interview participants

Table A1: Case study interview participants

Site	Orthopaedic surgeons	Nursing staff	Managers	External (e.g. CCG)	Total
1	3	2	3	3	11
2	2	0	7	0	9
3	5	1	1	0	7
4	5*	0	3	0	8
5	6	0	1	0	7
6	3	0	5	0	8
Total	24	3	20	3	50

*Included one spinal surgeon

Appendix 2: Case study topic guide

Topic guide development:

The topic guide for the provider interviews was initially developed through preliminary discussions with the GIRFT programme team (to understand the programme, its programme theory, and any perceived facilitators or barriers to implementation) and the wider research team (to ensure that questions about resource costs and implementation were incorporate to facilate the quantitative and economic analyses).

The draft provider topic guide was finalised in January 2017 and piloted at one of the case study sites. It worked well and was consequently used across all sites. The topic guide was then iteratively refined during the course of the study, in response to both our preliminary findings and the evolution of the GIRFT programme as it was rolled out across the country.

Summary topic guide for provider interviews:

- Awareness of GIRFT programme in orthopaedics
    - First aware of GIRFT?

- Involvement in a GIRFT visit to this site?

- First visit by the GIRFT team
    - Experiences - When, duration, preparation, views on datapacks, main issues raised - helpful/challenging?, expectations, differences to other programmes
    - Actions following first visit

- GIRFT re-visit
    - Prompts as above plus – differences from first visit

- Other local/national quality improvement initiatives
    - Level of involvement, adoption, impact; Model Hospital

- Impact of GIRFT
    - Organisational practice, timescales for changes to practice, changes to resource allocation, help from GIRFT team to deliver changes
    - Attribution to GIRFT
    - Unintended consequences

- Monitoring and measuring change – cost savings, NJR compliance, less low volume procedures, infection rates.

- Other local contextual information
    - Local coding, specific local difficulties in implementing GIRFT recommendations

- Suggestions for improvements to GIRFT programme

- Implementation of GIRFT recommendations since 1st visit (in relation to key metrics)
    - How facilitated, duration, barriers/facilitators
    - Any GIRFT recommendation particularly difficult to implement locally
    - Any local controversies about particular GIRFT recommendations locally
    - Impact of these

    - Monitoring and measuring progress against GIRFT recommendations
    - Attribution to GIRFT

- Awareness of recent changes to GIRFT programme
    - Involvement with implementation hubs; GIRFT national/regional involvement
