# Supplementary material for: Mixed methods evaluation of the Getting it Right First Time programme in elective orthopaedic surgery in England: an analysis from the National Joint Registry and Hospital Episode Statistics
Source: BMJ Open. 2022 Jun 16;12(6):e058316. doi: 10.1136/bmjopen-2021-058316 (PMC9207914; doi:10.1136/bmjopen-2021-058316)
Supplement: Author's manuscript [file bmjopen-2021-058316.draft_revisions.pdf]

BMJ Open

BMJ Open is committed to open peer review. As part of this commitment we make the peer review history of every article we publish publicly available.

When an article is published we post the peer reviewers' comments and the authors' responses online. We also post the versions of the paper that were used during peer review. These are the versions that the peer review comments apply to.

The versions of the paper that follow are the versions that were submitted during the peer review process. They are not the versions of record or the final published versions. They should not be cited or distributed as the published version of this manuscript.

BMJ Open is an open access journal and the full, final, typeset and author-corrected version of record of the manuscript is available on our site with no access controls, subscription charges or pay-per-view fees (http://bmjopen.bmj.com).

If you have any questions on BMJ Open's open peer review process please email info.bmjopen@bmj.com

BMJ Open

A mixed methods evaluation of the Getting it Right First Time programme in elective orthopaedic surgery in England: an analysis from the National Joint Registry and Hospital Episode Statistics

Journal:	*BMJ Open*
Manuscript ID	bmjopen-2021-058316
Article Type:	Original research
Date Submitted by the Author:	12-Oct-2021
Complete List of Authors:	Barratt, Helen; UCL, Department of Applied Health Research<br>Hutchings, Andrew; London School of Hygiene and Tropical Medicine<br>Pizzo, Elena; University College London, Applied Health Research<br>Aspinal, Fiona; University College London, Department of Applied Health Research<br>Jasim, Sarah; LSE, Care Policy & Evaluation Centre<br>Gafoor, Rafael; University College London, Department of Applied Health Research<br>Ledger, Jean; University College London, Department of Applied Health Research<br>Mason, James; University of Warwick, Warwick Medical School<br>Mehta, Raj; University College London, Department of Applied Health Research<br>Martin, Peter; University College London, Applied Health Research<br>Fulop, Naomi; University College London, Applied Health Research<br>Morris, Stephen; University of Cambridge, Primary Care Unit<br>Raine, Rosalind; University College London, Institute of Epidemiology & Health
Keywords:	Hip < ORTHOPAEDIC & TRAUMA SURGERY, Knee < ORTHOPAEDIC & TRAUMA SURGERY, SURGERY

SCHOLARONE™
Manuscripts

I, the Submitting Author has the right to grant and does grant on behalf of all authors of the Work (as defined in the below author licence), an exclusive licence and/or a non-exclusive licence for contributions from authors who are: i) UK Crown employees; ii) where BMJ has agreed a CC-BY licence shall apply, and/or iii) in accordance with the terms applicable for US Federal Government officers or employees acting as part of their official duties; on a worldwide, perpetual, irrevocable, royalty-free basis to BMJ Publishing Group Ltd ("BMJ") its licensees and where the relevant Journal is co-owned by BMJ to the co-owners of the Journal, to publish the Work in this journal and any other BMJ products and to exploit all rights, as set out in our licence.

The Submitting Author accepts and understands that any supply made under these terms is made by BMJ to the Submitting Author unless you are acting as an employee on behalf of your employer or a postgraduate student of an affiliated institution which is paying any applicable article publishing charge ("APC") for Open Access articles. Where the Submitting Author wishes to make the Work available on an Open Access basis (and intends to pay the relevant APC), the terms of reuse of such Open Access shall be governed by a Creative Commons licence – details of these licences and which Creative Commons licence will apply to this Work are set out in our licence referred to above.

Other than as permitted in any relevant BMJ Author's Self Archiving Policies, I confirm this Work has not been accepted for publication elsewhere, is not being considered for publication elsewhere and does not duplicate material already published. I confirm all authors consent to publication of this Work and authorise the granting of this licence.

For peer review only

A mixed methods evaluation of the Getting it Right First Time programme in elective orthopaedic surgery in England: an analysis from the National Joint Registry and Hospital Episode Statistics

Helen Barratt* - Clinical Senior Research Associate
Department of Applied Health Research, University College London, London, UK

Andrew Hutchings* - Assistant Professor
London School of Hygiene and Tropical Medicine, London, UK

Elena Pizzo - Principal Research Fellow
Department of Applied Health Research, University College London, London, UK

Fiona Aspinal - Senior Research Associate
Department of Applied Health Research, University College London, London, UK

Sarah Jasim - Senior Research Associate
Department of Applied Health Research, University College London, London, UK

Rafael Gafoor - Senior Research Associate
Department of Applied Health Research, University College London, London, UK

Jean Ledger - Senior Research Associate
Department of Applied Health Research, University College London, London, UK

Raj Mehta – Patient Advisor
Department of Applied Health Research, University College London, London, UK

James Mason - Professor of Health Economics
Warwick Medical School, University of Warwick, Coventry, UK

Peter Martin - Lecturer in Applied Statistics
Department of Applied Health Research, University College London, London, UK

Naomi J. Fulop - Professor of Health Care Organisation and Management
Department of Applied Health Research, University College London, London, UK

Steve Morris - RAND Professor of Health Services Research
Institute of Public Health, University of Cambridge, Cambridge, UK

Rosalind Raine - Professor of Health Care Evaluation
Department of Applied Health Research, University College London, London, UK

*Joint first author

Correspondence to
Dr Helen Barratt, Department of Applied Health Research, University College London, London WC1E 6BT, UK.
h. barratt@ ucl. ac. uk

For peer review only - http://bmjopen.bmj.com/site/about/guidelines.xhtml

Abstract

Objective: To evaluate the impact of the 'Getting it Right First Time' (GIRFT) national improvement programme in orthopaedic surgery.

Design: Mixed-methods study comprising statistical analysis of linked national datasets (National Joint Registry; Hospital Episode Statistics; Patient Reported Outcomes); economic (cost-consequences) analyses; and qualitative case studies in six NHS Trusts.

Setting: NHS elective orthopaedic surgery in England.

Participants: 736,088 patients who underwent a primary hip or knee replacement at 126 NHS Trusts between 1 April 2009-31 March 2018, plus 50 orthopaedic staff and Trust managers.

Intervention: Improvement bundle including 'deep dive' visits by a senior clinician to NHS Trusts, informed by a bespoke 'datapack' for each site, combining a range of routine performance data, to discuss where and how improvements could be made locally.

Main outcome measures: Number of procedures conducted by low volume surgeons; use of uncemented hip implants in patients >65; arthroscopy in year prior to knee replacement; hospital length of stay for index procedure; emergency readmissions within 30 days; revision surgery within one year; health-related quality of life; and functional status.

Results: National trends demonstrated substantial improvements beginning prior to GIRFT. Between 2012-2018, there were reductions in procedures by low volume surgeons (ORs (95% CI) hips 0.58 (0.53 to 0.63), knees 0.77 (0.72 to 0.83)); uncemented hip prostheses in over 65s (OR 0.56 (0.51 to 0.61)); knee arthroscopies before surgery (OR 0.48 (0.41 to 0.56)); and mean length of stay (hips -0.90 (-1.00 to -0.81), knees -0.74 days (-0.82 to -0.66)). The additional impact of visits was mixed. It differed between the earliest and later Trusts visited and comprised an overall economic saving of £431,848 between 2012-2018, but this was offset by the costs of the visits. Staff reported that GIRFT's influence ranged from procurement changes to improved regional collaboration.

Conclusion: At the national level, we found substantial improvements in care, but the specific contribution of GIRFT cannot be reliably estimated due to other concurrent national initiatives. Our approach enabled additional analysis of the discrete impact of GIRFT visits.

Strengths and limitations of this study

- We report the first, independent evaluation of the high profile Getting It Right First Time NHS improvement programme.
- Our mixed method approach enabled us to provide a comprehensive and robust understanding of GIRFT, exploring the impact of the programme from different perspectives.
- Our linked dataset allowed us to examine a range of measures, as well as estimating the specific contribution of Trust visits, whilst the case study analysis provided further insights from the perspective of Trust staff.
- We could not examine some key GIRFT target measures (e.g. procurement, litigation rates) because appropriate data were not available.

For peer review only - http://bmjopen.bmj.com/site/about/guidelines.xhtml

- We also could not capture costs incurred by Trusts, because activities were not consistently tracked.

Introduction

'Getting it Right First Time' (GIRFT) is one of the largest improvement programmes in the NHS. It began in orthopaedics in 2012 with the publication of the first GIRFT report, recommending changes to improve outcomes and reduce costs.[1] Following government investment totalling £62.8m, GIRFT now operates in 44 different specialities, or clinical workstreams.[2] GIRFT is an improvement 'bundle' - a small number of interventions performed together to improve care.[3] Clinical leadership is fundamental: GIRFT was established by a senior surgeon, who leads the orthopaedic workstream and now chairs the wider programme. Each workstream is chaired by a clinical lead from the relevant speciality. The programme includes components operating at local (i.e. NHS provider Trusts) and national (i.e. across England) levels. Local components include 'deep dive' visits to Trusts, whilst national components include national reports describing how unwarranted variations can be addressed. Initial first visits were piloted at a small number of Trusts in 2013, then replicated across the country in orthopaedics and other workstreams from 2015. Before each visit, Trusts are sent a bespoke 'datapack,' collated by GIRFT, describing their performance on over 100 variables, drawn from sources including Hospital Episode Statistics (HES) and national audits, such as the National Joint Registry (NJR). Data include use of evidence-based procedures and costs. Datapacks bring data sources together and make them accessible, facilitating comparison to national and peer group averages. Discussion at the meeting is driven by the datapack and tailored to the Trust. Attendees, comprising clinicians, managers and other relevant professionals, identify where and how improvements could be made. Revisits follow a similar format with Trusts reporting changes made since the first visit.

There are few examples of initiatives on this scale, and consequently few evaluations. The American College of Surgeons National Surgical Quality Improvement Program (NSQIP) provides data to drive improvement. However, only around 10% of US hospitals participate.[4] In the UK, the national Perioperative Quality Improvement Programme (PQIP) will collate and feedback data about surgical complications.[5] NHS Trusts are also subject to inspections by the Care Quality Commission, whilst some National Clinical Audits also provide written feedback.[6] However, GIRFT clinical leads visit all Trusts to discuss their datapack, as well as opportunities for improvement. GIRFT reflects evidence that measurement alone is insufficient.[7] The effectiveness of feedback is dependent on hard' and 'soft'[8] incentives, including using data to support change and holding participants to account at revisits.[9] However, although GIRFT visits Trusts twice, sometimes more, the largest improvements in NSQIP were seen where providers had participated for many years.[10] Improvements were also more marked at sites with pre-existing internal improvement programmes.[11] GIRFT makes use of system (top-down) leadership, but designated local (bottom-up) leadership within Trusts may also be important.[12] Despite the limited evidence base, GIRFT has been expanded across the NHS, in the absence of independent evaluation.

We therefore evaluated the effectiveness and cost-effectiveness of the GIRFT orthopaedic workstream, focussing on the most common elective procedures, primary hip and knee replacement.[13] The few evaluations of national programmes focus mainly on quantitative process and outcome measures, and hence may miss wider effects. Consequently we undertook a multicomponent analysis. The first described national trends in processes and outcomes over time, starting before GIRFT. However, this cannot disentangle the relative

For peer review only - http://bmjopen.bmj.com/site/about/guidelines.xhtml

impact of GIRFT from other concurrent national initiatives that targeted similar measures. For example, Lord Carter's review of NHS hospital efficiency took place between June 2014 and February 2016, and made recommendations about tackling 'unwarranted variation' in key resource areas such as staffing, diagnostics and procurement. NHS Right Care originated as part of the Quality, Innovation, Productivity and Prevention programme in 2009 and is still ongoing. It supports local economies to improve population health care and address performance variations. Our second analysis focused on a specific component of GIRFT, first visits to Trusts, to elucidate their *additional* local impact over and above the national trends. We also undertook an economic analysis to evaluate the cost impacts of visits, and a qualitative exploration of the impact of GIRFT from the perspective of Trust staff.

Methods

Design

We employed a mixed methods approach, using both quantitative and qualitative data to consider the various impacts of GIRFT from different perspectives and at different levels. (national and Trust). In the quantitative analysis, we examined eight key GIRFT target measures. We first describe changes in these at national level. We then exploit variation in the timing of GIRFT visits to assess the *additional* impact of initiating local involvement *after allowing for national trends*. We also undertook an economic analysis to assess costs and savings attributable to the visits. Finally, we used qualitative methods to explore how staff perceived that GIRFT had impacted practice at six case study sites.

Quantitative methods

Quantitative data sources

We used linked data from NJR, HES and Patient Reported Outcomes (PROMs) to identify patients aged 18 or over who underwent an elective primary hip or knee replacement between 1 April 2009-31 March 2018, at 126 English Trusts that received a first GIRFT visit by November 2017. We excluded eight Trusts visited later because they would contribute no post-visit data to the analysis. We included primary procedures eligible for the PROMs programme.[14] Metal-on-metal implants were excluded because they were subject to specific regulatory.[15]

Variables

We identified eight measures that were GIRFT priorities and available from the dataset. We were unable to use data on some priority indicators, such as litigation and procurement, because they are not publicly available. Process measures were: (i) procedures conducted by low volume surgeons (=<35 similar procedures or =< 10 unicondylar/ patella-femoral knee replacements per year); (ii) use of uncemented hip implants in patients >65; (iii) arthroscopy in year prior to knee replacement. GIRFT sought to reduce these, citing evidence that higher surgeon volumes equate to better outcomes; knee arthroscopy is not effective; and uncemented hip implants in older patients have higher revision rates.[1] Outcome measures comprised: (i) hospital length of stay for index procedure; (ii) emergency readmissions within 30 days; (iii) revision surgery within one year; and in the subset of patients who participated in the national PROMs programme (iv) health-related quality of life (EQ-5D)[16] and (v) functional status (Oxford Hip/ Knee Score (OHKS)).[17] GIRFT sought to minimise the first three to reduce associated costs. We included EQ5D and OHKS to facilitate economic evaluation. We identified revisions from NJR and HES,[18] measuring patient-reported outcomes at six

For peer review only - http://bmjopen.bmj.com/site/about/guidelines.xhtml

For peer review only

months.[14] We adjusted for: age in years; ethnicity (white, not white, or missing/not reported); sex; quintile of Index of Multiple Deprivation; Charlson comorbidity[19] (HES); and American Society of Anaesthesiologists grade (NJR).

Statistical analyses

We examined proportions and means of measures before GIRFT started (2009/10-2011/12), and in the final three years of the study (2015/16-2017/18). In our analysis of national trends, we also estimated year-on-year changes in comparison with 2012/13, the year GIRFT began, using casemix-adjusted hierarchical logistic and linear regression.

We analysed the additional impact of initiating visits using a pre-post design. First visit dates defined pre- and post- periods for Trusts. The order of visits was not random, so we divided Trusts into early, middle and late groups in a 1:2:1 ratio, to examine whether changes differed by visit timing. The ratio was specified *a priori* to split Trusts by lower and upper quartiles, ensuring a minimum of 30 per group. We used casemix-adjusted hierarchical logistic and linear regression models with clustering at Trust-level to estimate changes in levels for each measure. We controlled for temporal trends using fractional polynomials, and pre-operative scores in PROMs analyses. We used a complete case analysis but included a missing category for ethnicity. We allowed for implementation delays by excluding procedures in the three months after each initial Trust visit, based on a GIRFT case study indicating that improvements occured within three months.[20] We used a longer 15-month exclusion when analysing arthroscopies, because these were measured in the 12 months before surgery and hence might overlap the visit date.

Economic analysis methods

Conventionally, economic evaluation compares costs and outcomes of an intervention and comparator. However, it was not possible to use a comparator because GIRFT visited Trusts at different times. Instead, we compared the impact of visits at Trust level with expected costs in the absence of GIRFT. We examine: (1) cost of the visits; (2) costs incurred by Trusts to implement recommendations; and (3) costs or savings resulting from the visits in the limited measures publicly available for analysis. (Figure 1)

Information from the GIRFT programme team enabled us to calculate visit costs at Trust-level. We collected data on costs incurred by Trusts via a national survey, distributed electronically on our behalf, in early 2018, by the programme team, to GIRFT 'champions' in each Trust. It included questions about five GIRFT recommendations that could have an economic impact: implementation of ring-fenced beds; introduction of extended physiotherapy services; changes in use of theatre loan kits; reductions in activity by low volume surgeons; and improvements to theatre efficiency.

We assessed the economic impact of changes using the results of the Trust-level analysis above, quantified using NHS cost data.[21] We used the marginal effects estimated in the statistical models to estimate the economic impact of the visits where a statistically significant change was observed.

Qualitative methods

We used a case study approach to explore the implementation of GIRFT in individual NHS Trusts, including staff perceptions about whether and how GIRFT impacted practice.[22]

For peer review only - http://bmjopen.bmj.com/site/about/guidelines.xhtml

Case study sites

We purposively sampled six Trusts in England, representing a spread of hospital types (district general and teaching hospitals) and setting (region and rural versus urban).

Qualitative data collection

We have described data collection methods previously.[13] Here, we report relevant data collected via semi-structured interviews between Oct 2016-May 2019. Interviewees included surgeons and other staff present at the first GIRFT visit or knowledgeable about local improvement. Interviews lasted between 20-60 minutes and were audio-recorded for full transcription.

Qualitative data analysis

We analysed data thematically, within and across cases, combining inductive and deductive approaches.[23]

Patient and Public Involvement

From the inception of the study, we worked with the NIHR CLAHRC North Thames lay Research Advisory Panel to to refine the protocol and ensure that the proposed research appropriately reflected the priorities, experience, and preferences of patients. Through this, we identified a patient representative (RM) who agreed to join the study steering board. He has subsequently played a collaborative role in refining the research design, interpreting findings and disseminating results. The research reported here did not directly involve patients, so RM did not play a role in recruitment to the study.

Ethics approval

Granted by NHS North West - Liverpool East Research Ethics Committee (16/NW/0654).

Results

Quantitative findings

Study population

337,279 patients who underwent a hip replacement and 398,809 who received a knee replacement in 126 NHS Trusts, between 1 April 2009-31 March 2018. (Figures 2+3) See Table 1 for patient characteristics.

1) National trends

Process measures

Nationally, there were substantial improvements in process measures, often beginning before 2012 when GIRFT (Figures 4+5). Comparing 2009-2012 and 2015-2018, reductions varied from 29% fewer uncemented hips to a 58% reduction in knee arthroscopy. (Table 2) Reductions in procedures by low volume surgeons began prior to GIRFT for hips and knees. Odds ratios for 2017/18 versus 2012/13 were 0.58 (95% CI 0.53, 0.63) for hips and 0.77 (95% CI 0.72, 0.83)

For peer review only - http://bmjopen.bmj.com/site/about/guidelines.xhtml

For peer review only

for knees. Reductions in uncemented hips and knee arthroscopy also began prior to 2012, but the largest occurred as GIRFT progressed with odds ratios for 2017/18 versus 2012/13 of 0.56 (95% CI 0.51, 0.61) and 0.48 (95% CI 0.41, 0.56) respectively.

Outcome measures

Mean length of stay reduced by just over one day for both hip and knee patients between 2009-2012 and 2015-2018. The largest reductions began before GIRFT (Figures 4+5). Mean differences between 2012/13 and 2017/18 were -0.90 days (95% CI -1.00, -0.81) for hips and -0.77 days (95% CI -0.82, -0.66) for knees. There was some evidence that one-year knee revisions declined by 2017/18, but little evidence of a change in hips. Post-operative quality of life and functional status improved, but there was little change in emergency readmissions (Table 2 and Figures 4+5).

2) Additional effect of initiating 'deep dive' involvement at individual Trusts

GIRFT's first visits to Trusts occurred between September 2013 and November 2017 with half occurring between February and July 2014. (Table 3)

Process measures

The additional effects of the visits on process measures, after controlling for national trends, differed between the earliest Trusts visited and the middle and later groups. In the earliest group, we observed reductions in procedures by low volume surgeons. Conversely, this increased in the middle and late groups, but use of uncemented hips reduced. (Table 3)

Outcome measures:

Following visits, mean length of stay increased for the middle and late groups, but decreased in hip patients in the earlier group. However, estimated effects were small in comparison with national trends. We found limited evidence of an impact on other outcomes. (Table 3).

Economic analysis

The estimated cost to deliver the Trust visits was £491,420 (Component 1). This is a 'sunk' cost, including £10,769 transport costs (total return trip mileage); £150,000 for datapacks; £111,916 GIRFT staff time; and £106,820 opportunity cost of Trust staff time attending the meetings.

Our national survey provided mixed reports about the implementation of recommendations so it was not possible to accurately estimate costs. Therefore, although Trusts incurred costs, these could not be included (Component 2).

Although length of stay increased after the visits ($p<0.05$), this was not included in our economic analysis. Trusts receive the same payment for each inpatient stay, up to the tariff 'trim point' (currently nine days). However, average length of stay remained below this, despite the observed increase, so there was no extra monetary cost. Nevertheless, increased length of stay does come with an opportunity cost, as the bed is not available for another patient. As there was no change in EQ-5D, used to calculate quality adjusted life years, we were unable to undertake a cost-effectiveness analysis. Instead, we conducted a cost-consequences analysis. We observed positive and negative changes after GIRFT visits (Table 3) and additional costs partly outweighed savings (Table 4). The overall impact of the

For peer review only - http://bmjopen.bmj.com/site/about/guidelines.xhtml

statistically significant changes, for the limited number of measurable variables, not including length of stay, was a saving of £431,848 (Component 3).

Case-study analysis

We conducted 50 interviews across six sites (Appendix). Interviewees described five types of impact, operating at three levels: individual, Trust and regional (Figure 6). These ranged from changes to implant selection, to improved networking within Trusts and across regions. However, GIRFT particularly impacted ways of working at Trust-level, for example, catalysing planned improvements.

Interaction within and between the three levels of impact was key. For example, reducing low volume surgery depended on regional partners forming networks. Similarly, increasing the number of procedures conducted per day required within-organisation negotiation to access theatre time. Visits were most successful when GIRFT aligned with Trust priorities (e.g. rationalising procurement - Site 3). They were less effective where GIRFT measures were not a local priority because of more immediate demands, for example, financial pressures

A range of factors, in addition to GIRFT, impacted practice. These included the concurrent roll out of other initiatives. For example, the Carter Review impacted procurement (Sites 2 and 6), whilst reductions in arthroscopies were driven by the Payment by Results Assurance Framework (Site 2).

Discussion

This is the first independent evaluation of GIRFT. We found substantial improvements in orthopaedic care during the first six years of the programme, notably reductions in uncemented hip prostheses, knee arthroscopies and length of stay. However, these started before GIRFT. It was not possible to estimate the distinct contribution of the programme, because of other concurrent initiatives with common goals (e.g. Carter Review). We also estimated the additional impact of GIRFT visits. We found a mix of positive and negative effects, generally small compared to overall improvements and differing between the earliest and latest Trusts visited. It is important to note, though, that the eight measures we analysed in our quantitative analyses, and targeted by GIRFT, relate to direct patient care. Staff at our case study sites reported that the programme had had an impact, but the effects that they described related much more to ways of working at Trust-level (e.g. improved networking) rather than direct patient care.

Our mixed method approach has enabled us to provide a comprehensive and robust understanding of GIRFT, using quantitative and qualitative data to explore the impact of the programme from different perspectives . Previous similar evaluations tend to focus on quantitative analyses. Our linked dataset allowed us to examine a range of measures, as well as estimating the specific contribution of Trust visits. The case study analysis provided further insights from the perspective of Trust staff. However, we measured changes at three months post-visit, and although some made improvements within this window, it may not have been sufficient for others. A further limitation is that we could not examine other key target measures. Procurement data were only available for 2017-19 and could not be compared to previous years. Although litigation data are available, our previous work[24] demonstrates a significant lag from incident to resolution. Consequently, it would not be possible to determine whether changes were an impact of GIRFT or other policies (e.g. Sign up to Safety). Other outcomes, such as 5- and 10-year revisions, were beyond our time frame, as were impacts after 2018. We also could not capture costs incurred by Trusts, because activities

For peer review only - http://bmjopen.bmj.com/site/about/guidelines.xhtml

were not consistently tracked. Finally, our qualitative interviews took place several months after the first GIRFT visits, creating a risk of recall bias.[25]

In 2020, the GIRFT team published an internal evaluation[20] describing how the orthopaedic workstream had supported Trusts to release 'financial opportunities' of £696million. Our findings differ for a number of reasons. First the internal evaluation was limited to descriptive analyses of national trends between 2013/14 and 2018/19. This is broadly consistent with our national trend analysis, although we adjusted for casemix and included data from 2009 to explore changes prior to GIRFT. In contrast, the GIRFT team attributed all trend changes to the programme. Second, they limited their economic analysis to the impact on processes and outcomes, whereas we also examined the cost of the visits and costs incurred by Trusts. Finally, our diverse case study sites facilitated cross-case comparison, to create a detailed contextual picture. The internal evaluation includes individual case studies which exemplify success. Early narrative reviews of GIRFT were published by the Kings Fund[26] and NHS Providers[27] However, these are not formal evaluations.

Our finding of improvements in processes but less clear changes in outcomes is consistent with evidence that improvement initiatives generally have greater impact on processes of care than patient outcomes.[28] Improvements observed before 2012 may be explained by GIRFT identifying existing best practice to share more widely. The additional impact of visits was mixed and, in some cases, worsened immediately afterwards. However, visits were just one part of the programme. Other components, such as national reports, also impacted care at case study sites. There may also be differences between Trusts visited earlier and later. One possible explanation is that later cohorts made changes prior to their visit secondary to information disseminated by Trusts visited earlier. However, our findings reflect other literature illustrating the challenges for improvement programmes in outperforming the secular trend, including the possibility that the programmes themselves may be implicated in that trend.[29]

GIRFT is one of the largest improvement initiatives in the NHS. Our analysis demonstrates significant improvements in orthopaedic care over the past 10 years, likely attributable to both GIRFT and other concurrent initiatives. The additional impact of visits was mixed. Given the substantial cost and expansion of the programme, ongoing monitoring and access to additional relevant data, including details of Trust activities, is recommended to enhance the ability to hold GIRFT and other national improvement progammes to account.

For peer review only

Contributors

HB was principal investigator. HB and RR initiated the research. HB, AH, EP, NJF, SM and RR designed the evaluation. SJ, AH and RG created the linked dataset. RG and AH conducted the quantitative analyses; PM provided statistical advice. SJ and EP designed the Trust survey and analysed the resultant data. EP and SM led the economic analysis. SJ and JL were responsible for qualitative data collection; FA, JL, SJ, HB and NJF analysed the qualitative data. HB, AH, EP, FA and RR drafted the manuscript and all authors contributed to reviewing and revision. All authors approved the final version. All authors had full access to all the data in the study and accept responsibility to submit for publication. HB will act as guarantor.

Declaration of interests

All authors have completed the ICMJE uniform disclosure form at www.icmje.org/

For peer review only - http://bmjopen.bmj.com/site/about/guidelines.xhtml

coi_disclosure.pdf and declare: HB, AF, EP, FA, SJ, RG, JL, and RR are funded in full or in part by the National Institute for Health Research ARC North Thames and NJF is an NIHR Senior Investigator; no financial relationships with any organisations that might have an interest in the submitted work in the previous three years; no other relationships or activities that could appear to have influenced the submitted work. The views expressed in this publication are those of the authors and not necessarily those of the National Institute for Health Research or the Department of Health and Social Care.

Transparency declaration

The corresponding author (the manuscript's guarantor) affirms that the manuscript is an honest, accurate, and transparent account of the study being reported; that no important aspects of the study have been omitted; and that any discrepancies from the study as originally planned have been explained.

Role of the funder

The funder had no role in study design, data collection, data analysis, data interpretation, or writing of the report.

Data sharing

In line with the data sharing agreements between University College London and NHS Digital/ HQIP, aggregate small number suppressed outputs for the study period (1 April 2009-31 March 2018) are available on request from the corresponding author, Dr Helen Barratt (h.barratt@ucl.ac.uk).

Licence statement

I, the Submitting Author has the right to grant and does grant on behalf of all authors of the Work (as defined in the author licence), an exclusive licence and/or a non-exclusive licence for contributions from authors who are: i) UK Crown employees; ii) where BMJ has agreed a CC-BY licence shall apply, and/or iii) in accordance with the terms applicable for US Federal Government officers or employees acting as part of their official duties; on a worldwide, perpetual, irrevocable, royalty-free basis to BMJ Publishing Group Ltd ("BMJ") its licensees.

The Submitting Author accepts and understands that any supply made under these terms is made by BMJ to the Submitting Author unless you are acting as an employee on behalf of your employer or a postgraduate student of an affiliated institution which is paying any applicable article publishing charge ("APC") for Open Access articles. Where the Submitting Author wishes to make the Work available on an Open Access basis (and intends to pay the relevant APC), the terms of reuse of such Open Access shall be governed by a Creative Commons licence – details of these licences and which licence will apply to this Work are set out in our licence referred to above.

Acknowledgements

We thank Patricia Hallam for administrative support for the study. We thank the patients and staff of all the hospitals in England, Wales and Northern Ireland who have contributed data to the National Joint Registry. We are grateful to the Healthcare Quality Improvement Partnership (HQIP), the NJR Research Committee and staff at the NJR Centre for facilitating this work. The authors have conformed to the NJR's standard protocol for data access and publication. The views expressed represent those of the authors and do not necessarily reflect

For peer review only - http://bmjopen.bmj.com/site/about/guidelines.xhtml

those of the National Joint Registry Steering Committee or the Healthcare Quality Improvement Partnership (HQIP) who do not vouch for how the information is presented. This report is independent research funded by the National Institute for Health Research ARC North Thames. The views expressed in this publication are those of the authors and not necessarily those of the National Institute for Health Research or the Department of Health and Social Care.

For peer review only

For peer review only - http://bmjopen.bmj.com/site/about/guidelines.xhtml

Tables

Table 1: Characteristics of patient population*

	Hip replacement patients (n = 337,279)		Knee replacement patients (n = 398,809)	
	**N**	**%**	**N**	**%**
**Age**				
10 – 19	37	0.0%	-	-
20 – 29	1,553	0.5%	140	0.0%
30 – 39	4,805	1.4%	851	0.2%
40 – 49	16,765	5.0%	10,443	2.6%
50 – 59	46,753	13.9%	53,736	13.5%
60 – 69	96,371	28.6%	131,197	32.9%
70 – 79	115,840	34.4%	144,271	36.2%
80 – 89	51,900	15.4%	55,873	14.0%
90 and over	3,255	1.0%	2,298	0.6%
**Sex**				
Male	136,468	40.5%	169,959	42.6%
Female	200,811	59.5%	228,850	57.4%
**Ethnicity**				
White	317,671	94.4%	360,742	90.5%
Other	6,941	2.2%	27,327	6.9%
Missing	12,667	3.8%	10,740	2.7%
**Index of multiple deprivation quintile**				
Least Deprived	78,059	23.1%	84,401	21.2%
Less Deprived	80,505	23.9%	88,714	22.2%
Deprived	61,831	18.3%	77,281	19.4%
More Deprived	59,340	17.6%	75,173	18.9%
Most Deprived	56,520	16.8%	72,153	18.1%
Missing	1,024	0.3%	1,087	0.3%
**Charlson comorbidity score**				
0	210,385	62.4%	232,953	58.4%
>= 1	126,894	37.6%	165,856	41.6%
**American Society of Anaesthesiologists Score**				
Healthy	40,379	12.0%	32,329	8.1%
Mild Systemic	227,471	67.4%	284,064	71.2%
Severe/Moribund	69,429	20.6%	82,416	20.7%

†IMD (Index of Multiple Deprivation) |‡ ASA (American Society of Anaesthesiologists Score) | ¥OHKS Oxford Hip Knee Score

For peer review only - http://bmjopen.bmj.com/site/about/guidelines.xhtml

Table 2: Process and outcome measures across the study population at the beginning and end of the study period, 1 Apr 2009-31 Mar 2018

		**Beginning of study period** (01/04/09-31/12/12)	**End of study period** (01/04/16-31/03/18)
**Process measures**			
Low volume surgery (<35 per annum) (%)	Hip	15.9% (95% CI 15.70 to 16.2)	9.1% (95% CI 8.9 to 9.3)
	Knee	18.6% (95% CI 18.4 to 18.8)	13.4% (95% 13.2 to 13.7)
Uncemented hip implant in >65s (%)		31.7% (95% CI 31.4 to 32.1)	22.6% (95% CI 22.20 to 23.0)
Arthroscopy in previous 12m (%)		6.5% (95% CI 6.3 to 6.56)	3.4% (95% CI 3.3 to 3.5)
**Outcome measures**			
Length of stay (median and mean days)	Hip	5 (IQR 3 to 6) 5.6 (95% CI 5.6 to 5.6)	3 (IQR 2 to 5) 4.3 (95% CI 4.3 to 4.4)
	Knee	5 (IQR 3 to 6) 5.5 (95% CI 5.5 to 5.5)	4 (IQR 3 to 5) 4.3 (95% CI 4.3 to 4.4)
Readmissions in 30 Days (%)	Hip	4.7% (95% CI 4.5 to 4.8)	4.6% (95% CI 4.4 to 4.7)
	Knee	5.2% (95% CI 5.1 to 5.3)	5.0% (95% CI 4.9 to 5.2)
Revisions in 12 Months (%)	Hip	0.77% (95% CI 0.72 to 0.82)	0.63% (95% CI 0.58 to 0.69)
	Knee	0.44% (95% CI 0.40 to 0.48)	0.37% (95% CI 0.33 to 0.41)
Post-operative Oxford Hip/ Knee Score (mean)	Hip	38.1 (95% CI 38.0 to 38.2)	39.2 (95% CI 39.2 to 39.3)
	Knee	33.7 (95% CI 33.6 to 33.8)	35.3 (95% CI 35.2 to 35.4)
Post-operative EQ5D score (mean)	Hip	0.76 (95% CI 0.76 to 0.77)	0.78 (95% CI 0.78 to 0.78)
	Knee	0.70 (95% CI 0.70 to 0.70)	0.73 (95% CI 0.73 to 0.73)

For peer review only - http://bmjopen.bmj.com/site/about/guidelines.xhtml

Table 3: Changes in process and outcome measures after first GIRFT visit by Trust cohort*
(Statistically significant values in bold)

		**Early cohort**<br>10/09/13-02/02/14<br>(n=31 Trusts)	**Middle cohort**<br>03/02/14-06/07/14<br>(n=61 Trusts)	**Late cohort**<br>07/07/14-27/11/17<br>(n=34 Trusts)
**Process measures** (ORs below 1 indicate improvement)				
Low volume surgery (<35 per annum) (OR)	Hip	**0.92<br>(95% CI 0.87 to 0.98)**	**1.12<br>(95% CI 1.06 to 1.18)**	**1.11<br>(95% CI 1.04 to 1.19)**
	Knee	**0.92<br>(95% CI 0.87 to 0.97)**	**1.13<br>(95% CI 1.08 to 1.19)**	**1.19<br>(95% CI 1.13 to 1.26)**
Uncemented hip implant in >65s (OR)		1.07<br>(95% CI 1.00 to 1.16)	**0.82<br>(95% CI 0.77 to 0.87)**	**0.73<br>(95% CI 0.69 to 0.78)**
Arthroscopy in previous 12m (OR)		1.09<br>(95% CI 0.99 to 1.20)	1.08<br>(95% CI 0.99 to 1.18)	**1.19<br>(95% CI 1.06 to 1.33)**
**Outcome measures** (Negative change in length of stay, ORs below 1, and positive changes in Oxford Hip/Knee and EQ5D scores indicate improvement)				
Length of stay (change in mean days)	Hip	**-0.14<br>(95% CI -0.20 to -0.07)**	**0.09<br>(95% CI 0.03 to 0.14)**	**0.11<br>(95%CI 0.04 to 0.18)**
	Knee	-0.01<br>(95% CI -0.06 to 0.04)	**0.07<br>(95% CI 0.03 to 0.12)**	**0.06<br>(95% CI 0.01 to 0.12)**
Readmissions within 30 days (OR)	Hip	1.01<br>(95% CI 0.92 to 1.10)	1.07<br>(95% CI 0.99 to 1.16)	1.03<br>(95% CI 0.94 to 1.13)
	Knee	1.08<br>(95% CI 0.99 to 1.17)	1.03<br>95% CI (0.96 to 1.11)	**1.16<br>(95% CI 1.06 to 1.26)**
Revisions within 12 months (OR)	Hip	1.14<br>(95%CI 0.91 to 1.44)	1.06<br>(95% 0.87 to 1.30)	1.16<br>(95% CI 0.92 to 1.47)
	Knee	1.14<br>(95% CI 0.94 to 1.38)	0.95<br>(95% 0.82 to 1.10)	0.84<br>(95% CI 0.68 to 1.04)
Oxford Hip/ Knee Score (change in mean score)	Hip	**-0.12<br>(95% CI -0.33 to -0.09)**	-0.17<br>(95% CI -0.35 to 0.01)	-0.27<br>(95% CI -0.48 to 0.05)
	Knee	**-0.37<br>(95% CI -0.58 to -0.15)**	**-0.34<br>(95% CI -0.53 to -0.16)**	**-0.37<br>(95% CI -0.59 to -0.14)**
EQ-5D (change in mean score)	Hip	0.00<br>(95% CI 0.00 to 0.01)	0.00<br>(95% CI 0.00 to 0.01)	0.00<br>(95% CI 0.00 to 0.00)
	Knee	0.00<br>(95% CI-0.01 to 0.01)	0.00<br>(95% CI-0.01 to 0.01)	-0.01<br>(95% CI-0.01 to 0.00)

*Odds Ratios and linear regression coefficients adjusted for patient baseline characteristics and time variables

For peer review only - http://bmjopen.bmj.com/site/about/guidelines.xhtml

Table 4: Summary of GIRFT economic impact (2019/2020 GBP)

(Positive values are costs; negative values are savings; statistically significant values are in bold)

		Early cohort 10/09/13-02/02/14 (n=31 Trusts)	Middle cohort 03/02/14-06/07/14 (n=61 Trusts)	Late cohort 07/07/14-27/11/17 (n=34 Trusts)	Total results for all cohorts
**Process measures**					
Uncemented hip implant in >65s		87,654	**-444,107****	**-357,151****	- 713,604
Arthroscopy in previous 12m		242,365	330,927	**320,467****	893,759
**Outcome measures**					
Length of stay	Hip	-198,320	**5,641,544****	**2,513,717****	7,956,941
	Knee	**1,048,798****	**5,555,429****	**2,333,021****	8,937,249
Readmissions within 30 days	Hip	3,332	37,799	7,364	48,495
	Knee	37,325	21,785	**48,943****	108,053
Revisions within 12 months	Hip	411,777	290,035	327,938	1,029,750
	Knee	305,944	-182,231	-238,211	- 114,498
**Totals**					
**Overall economic impact[a]**		**1,938,876**	**11,251,181**	**4,956,088**	**18,146,144**
Savings [a]		-198,320	-626,338	-595,362	- 1,420,020
Incremental cost [a]		2,137,195	11,877,519	5,551,450	19,566,164
**Statistically significant economic impact [b]**		1,048,798	10,752,866	4,858,997	16,660,661
**Statistically significant economic impact (excluding LOS)[b]**		-	**-444,107****	**12,259****	**- 431,848****

[a] This is the sum of economic impact (saving or incremental cost) irrespective of statistically significant results.

[b] This is the sum of the economic impact taking into account only statistically significant results. ** statistically significant results at the 5% level

For peer review only - http://bmjopen.bmj.com/site/about/guidelines.xhtml

Figure legends

Figure 1: The economic components of GIRFT orthopaedic evaluation

Figure 2: Study population – total hip replacement patients
Patient Reported Outcomes Measures (PROMS) data collected from April 2009 for consenting patients only

Figure 3: Study population – total knee replacement patients
Patient Reported Outcomes Measures (PROMS) data collected from April 2009 for consenting patients only

Figure 4: Trends in process and outcome measures for primary hip replacements (2009/10-2017/18)

Figure 5: Trends in process and outcome measures for primary knee replacements (2009/10-2017/18)

Figure 6: Summary of impacts attributed to GIRFT orthopaedic visits at six case-study site

For peer review only

For peer review only - http://bmjopen.bmj.com/site/about/guidelines.xhtml

Appendix

Table A1: Case study interview participants

Site	Orthopaedic surgeons	Nursing staff	Managers	External (e.g. CCG)	Total
1	3	2	3	3	11
2	2	0	7	0	9
3	5	1	1	0	7
4	5*	0	3	0	8
5	6	0	1	0	7
6	3	0	5	0	8
Total	24	3	20	3	50

*Includes one spinal surgeon

For peer review only

References

1. Briggs T. *Getting it Right First Time: improving the quality of orthopaedic care within the National Health Service in England*. London: British Orthopaedic Association; 2012.

2. Briggs T. *Getting it Right First Time: a national review of adult elective orthopaedic services in England*. London: British Orthopaedic Association; 2015.

3. Resar R, Pronovost P, Haraden C, Simmonds T, Rainey T, Nolan T. Using a bundle approach to improve ventilator care processes and reduce ventilator-associated pneumonia. *J Qual Patient Saf*. 2005;31(5):243-8.

4. Maggard-Gibbons M. The use of report cards and outcome measurements to improve the safety of surgical care: the American College of Surgeons National Surgical Quality Improvement Program *BMJ Quality & Safety* 2014;**23:**589-599.

5. Peden CJ, Moonesinghe SR. Measurement for improvement in anaesthesia and intensive care. *Br J Anaesth*. 2016;117(2):145-148.

6. Khan T, Alderson SA, Francis JJ, Lorencatto F, Grant-Casey J, et al. Repeated analyses of national clinical audit reports demonstrate improvements in feedback methods. Implementation Science Communications 2020 ; 1 (1) :1-6

7. Berwick DM. Measuring Surgical Outcomes for Improvement: Was Codman Wrong? *JAMA.* 2015;313(5):469–470. doi:10.1001/jama.2015.4

8. Dixon-Woods M, Bosk CL, Aveling EL, Goeschel CA, Pronovost PJ. Explaining Michigan: developing an ex post theory of a quality improvement program. Milbank Q. 2011;89:167–205.

9. Dixon-Woods M, McNicol S, Martin G. Ten challenges in improving quality in healthcare: lessons from the Health Foundation's programme evaluations and relevant literature. *BMJ Quality & Safety* 2012;**21:**876-884.

10. Cohen ME, Liu Y, Ko CY, Hall BL. Improved Surgical Outcomes for ACS NSQIP Hospitals Over Time: Evaluation of Hospital Cohorts With up to 8 Years of Participation. Ann Surg. 2016 Feb;263(2):267-73. doi: 10.1097/SLA.0000000000001192. PMID: 25723845.

11. Montroy J, Breau RH, Cnossen S, Witiuk K, Binette A, Ferrier T, Lavallée LT, Fergusson DA, Schramm D. Change in Adverse Events After Enrollment in the National Surgical Quality Improvement Program: A Systematic Review and Meta-Analysis. PLoS One. 2016 Jan 26;11(1):e0146254. doi: 10.1371/journal.pone.0146254. PMID: 26812596; PMCID: PMC4727780.

12. Turner S, Ramsay A, Perry C, Boaden R, McKevitt C, Morris S, et al. Lessons for major system change: centralization of stroke services in two metropolitan areas of England. *J Health Serv Res Policy*. 2016;21:156–65.

13. Barratt H, Turner S, Hutchings A, Pizzo E, Hudson E, Briggs T, et al. Mixed methods evaluation of the Getting it Right First Time programme – improvements to NHS orthopaedic care in England: study protocol. *BMC Health Serv Res* 2017;17:71

For peer review only - http://bmjopen.bmj.com/site/about/guidelines.xhtml

14. NHS Digital. Patient Reported Outcome Measure https://digital.nhs.uk/data-and-information/data-tools-and-services/data-services/patient-reported-outcome-measures-proms (Accessed 8 Jun 2021)

15. Matharu G S, Judge A, Pandit H G, Murray D W. Follow-up for patients with metal-on-metal hip replacements: are the new MHRA recommendations justified? *BMJ* 2018; 360 :k566 doi:10.1136/bmj.k566

16. EuroQol Research Foundation. EQ-5D-3L User Guide, 2018. Available from: https://euroqol.org/publications/user-guides (Accessed 8 Jun 2021)

17. Dawson J, Fitzpatrick R, Carr A, Murray D. Questionnaire on the perceptions of patients about total hip replacement. J Bone Joint Surg Br. 1996 Mar;78(2):185-90.

18. Sibanda N, Copley LP, Lewsey JD, et al. Revision rates after primary hip and knee replacement in England between 2003 and 2006, *PLoS Med*, 2008;5:e179

19. Armitage JN van der Meulen JH . Identifying co-morbidity in surgical patients using administrative data with the Royal College of Surgeons Charlson Score. *Br J Surg* 2010; 97 : 772 – 81

20. GIRFT. *Getting It Right in Orthopaedics. Reflecting on success and reinforing improvement: a follow-up on the GIRFT national specialty report on orthopaedics.* London: Getting it Right First Time, 2020. https://gettingitrightfirsttime.co.uk/wp-content/uploads/2020/02/GIRFT-orthopaedics-follow-up-report-February-2020.pdf (Accessed 29 April 2021)

21. NHS Improvement. NHS 2018/19 National Tariff: currencies and prices. https://improvement.nhs.uk/resources/national-tariff (Accessed 8 Jun 2021)

22. Yin RK. Validity and generalization in future case study evaluations. *Evaluation*. 2013;19:321–32

23. Bradley EH, Curry LA, Devers KJ. Qualitative data analysis for health services research: developing taxonomy, themes, and theory. *Health Serv Res*. 2007; 42:1758–72.

24. https://resolution.nhs.uk/wp-content/uploads/2017/08/UCL-final-evaluation-report-bid-scheme.pdf (Accessed 8 Jun 2021)

25. Sedgwick P. What is recall bias? *BMJ* 2012; 344 :e3519 doi:10.1136/bmj.e351

26. Timmins N. *Tackling variations in clinical care: Assessing the Getting It Right First Time (GIRFT) programme*. London: Kings Fund, 2017 https://www.kingsfund.org.uk/sites/default/files/field/field_publication_file/Getting_it_right_Kings_Fund_June_2017.pdf (Accessed 29 April 2021)

27. NHS Providers. *The Getting it Right First Time programme: Early views from the provider sector.* London: NHS Providers, 2018. https://nhsproviders.org/media/4365/girft-final-pdf.pdf (Accessed 29 April 2021)

28. Ivers N, Jamtvedt G, Flottorp S, Young JM, Odgaard-Jensen J, French SD, O'Brien MA, Johansen M, Grimshaw J, Oxman AD. Audit and feedback: effects on professional practice and healthcare

For peer review only - http://bmjopen.bmj.com/site/about/guidelines.xhtml

outcomes. Cochrane Database Syst Rev. 2012 Jun 13;(6):CD000259. doi: 10.1002/14651858.CD000259.pub3. PMID: 22696318.

29. Bion J, Richardson A, Hibbert P THE MATCHING MICHIGAN COLLABORATION & WRITING COMMITTEE*, et al* 'Matching Michigan': a 2-year stepped interventional programme to minimise central venous catheter-blood stream infections in intensive care units in England *BMJ Quality & Safety* 2013;**22:**110-123.

For peer review only

	What	How	Data source
**Cost of GIRFT visits**	Cost of GIRFT team<br>Data pack production<br>Cost of visits trips<br>Cost of visits time<br>Cost of Trusts staff time	Staff wages<br>Cost of data pack<br>Mileage cost x total miles<br>Transport/meeting time x wage/hr<br>Meetings time x wage/hr	NHS, PSSRU<br>GIRFT team data
**Cost for Trusts to implement recommendations**	Ring fenced<br>Extended physiotherapy<br>Theatre load kits<br>Minimum Volumes<br>Theatre Efficiency	National Survey to NHS Trusts	National Survey to NHS Trusts in August 2018
**Economic impact of changes in clinical practice and outcomes**	Arthroscopy procedures<br>Fixation methods<br>Readmissions<br>Revisions<br>Length of Stay<br>PROMS-QALYs	Quantitative analysis results<br>Volumes x costs of procedures	NJR and HES<br>National Tariffs<br>NICE reports<br>HRGs

Figure 1: The economic components of GIRFT orthopaedic evaluation

209x296mm (300 x 300 DPI)

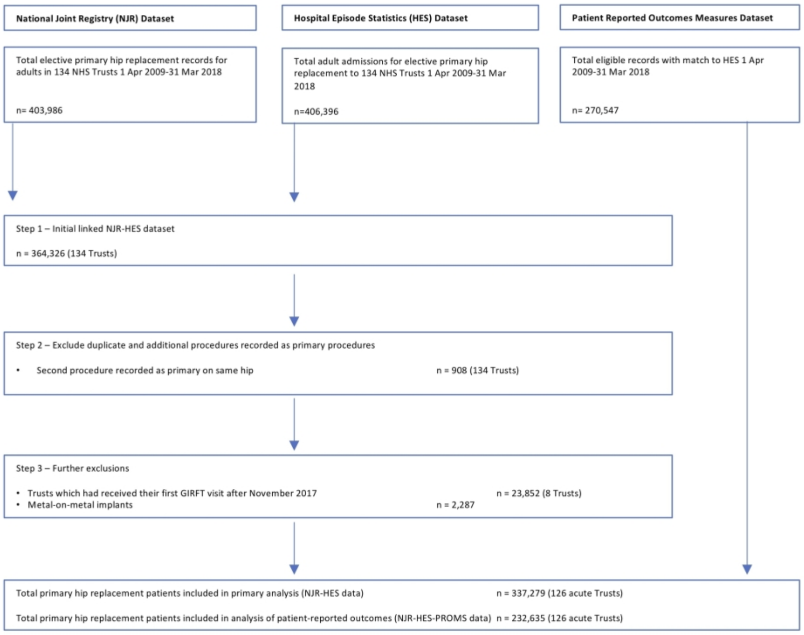

Figure 2: Study population – total hip replacement patients

190x254mm (300 x 300 DPI)

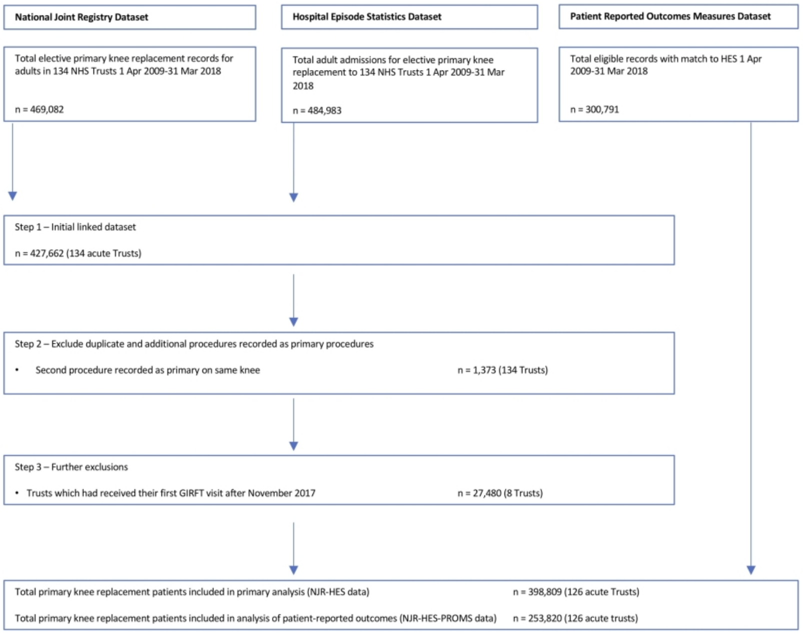

Figure 3: Study population – total knee replacement patients

190x275mm (300 x 300 DPI)

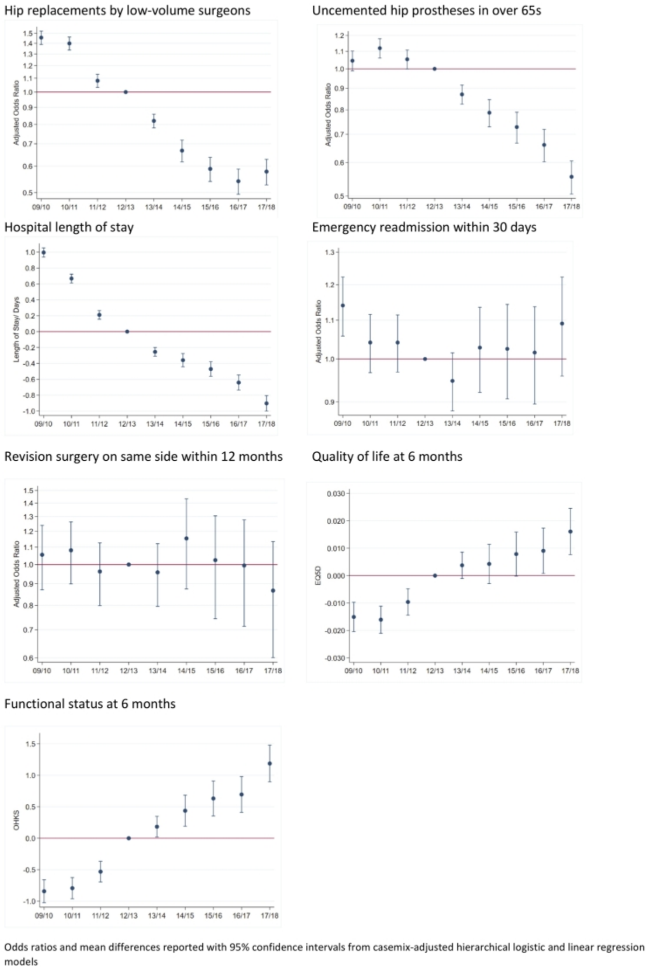

Figure 4: Trends in process and outcome measures for primary hip replacements (2009/10-2017/18)

209x296mm (300 x 300 DPI)

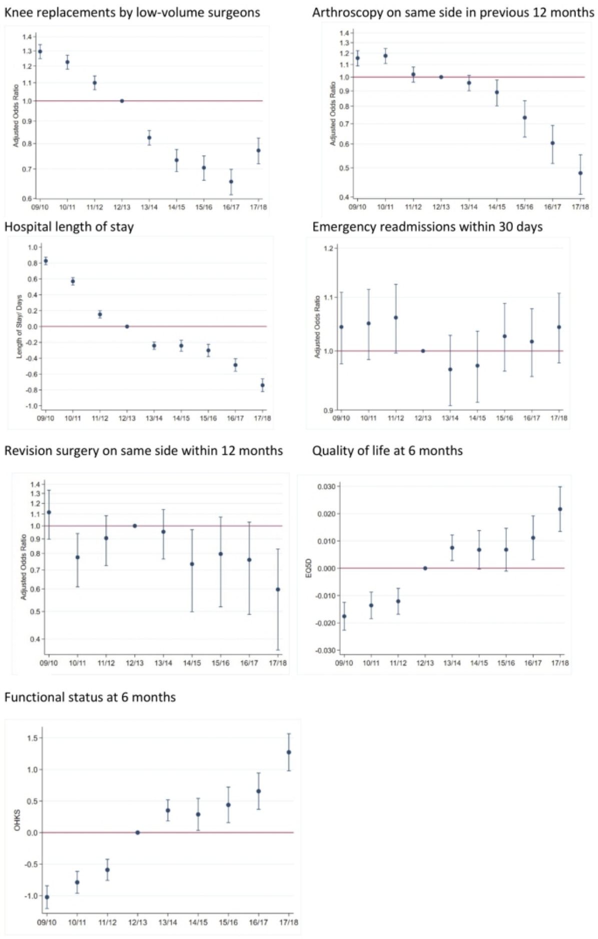

Odds ratios and mean differences reported with 95% confidence intervals from casemix-adjusted hierarchical logistic and linear regression models

Figure 5: Trends in process and outcome measures for primary knee replacements (2009/10-2017/18)

209x296mm (300 x 300 DPI)

Appendix

Table A1: Case study interview participants

Site	Orthopaedic surgeons	Nursing staff	Managers	External (e.g. CCG)	Total
1	3	2	3	3	11
2	2	0	7	0	9
3	5	1	1	0	7
4	5*	0	3	0	8
5	6	0	1	0	7
6	3	0	5	0	8
Total	24	3	20	3	50

*Includes one spinal surgeon

For peer review only

Level of impact	Type of impact	Examples
**Individual practitioner**	1. Changing clinical practice	Reduction in procedures conducted by low volume surgeons (Site 2)<br>Informed choices about implants (e.g. uncemented hips) (Sites 1+3)
**Trust**	2. Catalysing already planned changes	Expedited existing plans to improve implant selection and rationalisation of procurement (Site 3)
	3. Internal data review	Greater use of local data to inform decision-making (All sites)
	4. Influencing ongoing strategic conversations	Helped maintain focus on existing improvement agendas (e.g. ring fenced beds) (All)
	5. Increasing collaborative working	Local: introduction of weekly, multidisciplinary team review of cases (Site 3)
**Regional**		Regional: more networking with neighbouring Trusts (Sites 1+2)

Figure 6: Summary of impacts attributed to GIRFT orthopaedic visits at six case-study site

209x296mm (300 x 300 DPI)

STROBE Statement—Checklist of items that should be included in reports of ***cohort studies***

	Item No	Recommendation
**Title and abstract**	1	(*a*) Indicate the study's design with a commonly used term in the title or the abstract<br>**We describe the study as a mixed methods evaluation to indicate the study's overall design (title)**
		(*b*) Provide in the abstract an informative and balanced summary of what was done and what was found<br>**The abstract provides an overview of our methods and results.**
**Introduction**		
Background/rationale	2	Explain the scientific background and rationale for the investigation being reported<br>**We describe GIRFT (the 'intervention'), its context and the justification for the evaluation in the Introduction paragraphs 1-4.**
Objectives	3	State specific objectives, including any prespecified hypotheses<br>**We describe the objectives of the evaluation in the final paragraph of the Introduction.**
**Methods**		
Study design	4	Present key elements of study design early in the paper<br>**We include a summary of the overall design in the first (Design) paragraph of the Methods.**
Setting	5	Describe the setting, locations, and relevant dates, including periods of recruitment, exposure, follow-up, and data collection<br>**We describe the setting/locations/dates for the quantitative analysis in Methods (Quantitative methods: Data sources) and the case study sites (Qualitative methods: Case study sites and Data collection sections). Additional contextual information is provided in the Introduction.**
Participants	6	(*a*) Give the eligibility criteria, and the sources and methods of selection of participants. Describe methods of follow-up<br>**We report eligibility of NHS Trusts and patients for the quantitative analysis in Quantitative methods: Data sources and include information on the pre- and post-intervention data from Trusts. We provide a reference to details of selection of interviewees in Qualitative methods: data collection.**
		(*b*) For matched studies, give matching criteria and number of exposed and unexposed<br>**Not applicable**
Variables	7	Clearly define all outcomes, exposures, predictors, potential confounders, and effect modifiers. Give diagnostic criteria, if applicable<br>We report outcomes and potential confounders in Quantitative methods: Variables.<br>**We describe the 'exposure' as the period beginning with a Trust's first GIRFT visit in Quantitative methods: Statistical analysis as well as the potential modifying effect of the timing of the visit.**
Data sources/ measurement	8*	For each variable of interest, give sources of data and details of methods of assessment (measurement). Describe comparability of assessment methods if there is more than one group<br>**We report the routine data sources and definitions used in Quantitative methods: Data sources and Variables, which is the same for 'exposed' (post-) and 'unexposed' (pre-) periods in individual Trusts.**
Bias	9	Describe any efforts to address potential sources of bias<br>**We describe how we sought to control for temporal trends, non-random timing**

For peer review only

		**of GIRFT visits and 'washout' periods in Quantitative methods: Statistical analysis.**
Study size	10	Explain how the study size was arrived at<br>**The size of study was determined by availability of routine data rather than recruitment to a target sample size.**
Quantitative variables	11	Explain how quantitative variables were handled in the analyses. If applicable, describe which groupings were chosen and why<br>**We describe how independent variables were handled in Quantitative methods: Variables and how the Trusts were grouped by visit date in Quantitative methods: Statistical analysis.**
Statistical methods	12	(*a*) Describe all statistical methods, including those used to control for confounding<br>**We describe the statistical methods, including those used to control for confounding, in Quantitative methods: Statistical analysis.**
		(*b*) Describe any methods used to examine subgroups and interactions<br>**We describe the statistical methods, including those used to compare the different groups of NHS Trusts, in Quantitative methods: Statistical analysis.**
		(*c*) Explain how missing data were addressed<br>**We dealt with missing data using a complete case analysis. The exception was ethnicity where the missing/not reported category was included in the analysis. We report this in Quantitative methods: Variables and Statistical analysis and missing data are reported in Table 1.**
		(*d*) If applicable, explain how loss to follow-up was addressed<br>**Not applicable**
		(*e*) Describe any sensitivity analyses<br>**Not applicable**
**Results**		
Participants	13*	(a) Report numbers of individuals at each stage of study—eg numbers potentially eligible, examined for eligibility, confirmed eligible, included in the study, completing follow-up, and analysed
		(b) Give reasons for non-participation at each stage
		(c) Consider use of a flow diagram<br>**We have included flow diagrams (Figures 2+3), which set out the number of individuals included at each stage of the study (a), including reasons for non-participation (b).**
Descriptive data	14*	(a) Give characteristics of study participants (eg demographic, clinical, social) and information on exposures and potential confounders<br>**Table 1 describes the characteristics of study participants, including potential confounders.**
		(b) Indicate number of participants with missing data for each variable of interest<br>**This information is provided in Table 1.**
		(c) Summarise follow-up time (eg, average and total amount)<br>**Not applicable.**
Outcome data	15*	Report numbers of outcome events or summary measures over time<br>**We report summary measures of the outcome events over time in Table 2 and Figures 4+5**
Main results	16	(*a*) Give unadjusted estimates and, if applicable, confounder-adjusted estimates and their precision (eg, 95% confidence interval). Make clear which confounders were adjusted for and why they were included
		(*b*) Report category boundaries when continuous variables were categorized

For peer review only - http://bmjopen.bmj.com/site/about/guidelines.xhtml
For peer review only

		**This information is reported in Quantitative methods: variables, and also in Table 1.**
		(*c*) If relevant, consider translating estimates of relative risk into absolute risk for a meaningful time period **Not applicable.**
Other analyses	17	Report other analyses done—eg analyses of subgroups and interactions, and sensitivity analyses **Not applicable.**
**Discussion**		
Key results	18	Summarise key results with reference to study objectives **Key results are summarised in the first paragraph of the Discussion.**
Limitations	19	Discuss limitations of the study, taking into account sources of potential bias or imprecision. Discuss both direction and magnitude of any potential bias **Limitations of the study, including potential sources of bias, are listed in paragraph 2 of the Discussion.**
Interpretation	20	Give a cautious overall interpretation of results considering objectives, limitations, multiplicity of analyses, results from similar studies, and other relevant evidence **We provide an interpretation of the results in the penultimate paragraph of the Discussion. We set the findings in the context of other research in the third paragraph of the Discussion.**
Generalisability	21	Discuss the generalisability (external validity) of the study results **We discuss the generalisability of the results to other improvement initiatives in paragraphs 4+5 of the Discussion.**
**Other information**		
Funding	22	Give the source of funding and the role of the funders for the present study and, if applicable, for the original study on which the present article is based **We have included statements about the source of funding and the role of the funder in the author statements at the end of the article.**

*Give information separately for exposed and unexposed groups.

Note: An Explanation and Elaboration article discusses each checklist item and gives methodological background and published examples of transparent reporting. The STROBE checklist is best used in conjunction with this article (freely available on the Web sites of PLoS Medicine at http://www.plosmedicine.org/, Annals of Internal Medicine at http://www.annals.org/, and Epidemiology at http://www.epidem.com/). Information on the STROBE Initiative is available at http://www.strobe-statement.org.

For peer review only

For peer review only - http://bmjopen.bmj.com/site/about/guidelines.xhtml

BMJ Open

A mixed methods evaluation of the Getting it Right First Time programme in elective orthopaedic surgery in England: an analysis from the National Joint Registry and Hospital Episode Statistics

Journal:	*BMJ Open*
Manuscript ID	bmjopen-2021-058316.R1
Article Type:	Original research
Date Submitted by the Author:	15-Mar-2022
Complete List of Authors:	Barratt, Helen; UCL, Department of Applied Health Research<br>Hutchings, Andrew; London School of Hygiene and Tropical Medicine<br>Pizzo, Elena; University College London, Applied Health Research<br>Aspinal, Fiona; University College London, Department of Applied Health Research<br>Jasim, Sarah; LSE, Care Policy & Evaluation Centre<br>Gafoor, Rafael; University College London, Department of Applied Health Research<br>Ledger, Jean; University College London, Department of Applied Health Research<br>Mehta, Raj; University College London, Department of Applied Health Research<br>Mason, James; University of Warwick, Warwick Medical School<br>Martin, Peter; University College London, Applied Health Research<br>Fulop, Naomi; University College London, Applied Health Research<br>Morris, Stephen; University of Cambridge, Primary Care Unit<br>Raine, Rosalind; University College London, Institute of Epidemiology & Health
**Primary Subject Heading**:	Surgery
Secondary Subject Heading:	Health policy
Keywords:	Hip < ORTHOPAEDIC & TRAUMA SURGERY, Knee < ORTHOPAEDIC & TRAUMA SURGERY, SURGERY

SCHOLARONE™ Manuscripts

I, the Submitting Author has the right to grant and does grant on behalf of all authors of the Work (as defined in the below author licence), an exclusive licence and/or a non-exclusive licence for contributions from authors who are: i) UK Crown employees; ii) where BMJ has agreed a CC-BY licence shall apply, and/or iii) in accordance with the terms applicable for US Federal Government officers or employees acting as part of their official duties; on a worldwide, perpetual, irrevocable, royalty-free basis to BMJ Publishing Group Ltd ("BMJ") its licensees and where the relevant Journal is co-owned by BMJ to the co-owners of the Journal, to publish the Work in this journal and any other BMJ products and to exploit all rights, as set out in our licence.

The Submitting Author accepts and understands that any supply made under these terms is made by BMJ to the Submitting Author unless you are acting as an employee on behalf of your employer or a postgraduate student of an affiliated institution which is paying any applicable article publishing charge ("APC") for Open Access articles. Where the Submitting Author wishes to make the Work available on an Open Access basis (and intends to pay the relevant APC), the terms of reuse of such Open Access shall be governed by a Creative Commons licence – details of these licences and which Creative Commons licence will apply to this Work are set out in our licence referred to above.

Other than as permitted in any relevant BMJ Author's Self Archiving Policies, I confirm this Work has not been accepted for publication elsewhere, is not being considered for publication elsewhere and does not duplicate material already published. I confirm all authors consent to publication of this Work and authorise the granting of this licence.

For peer review only

A mixed methods evaluation of the Getting it Right First Time programme in elective orthopaedic surgery in England: an analysis from the National Joint Registry and Hospital Episode Statistics

Helen Barratt* - Clinical Senior Research Associate
Department of Applied Health Research, University College London, London, UK

Andrew Hutchings* - Assistant Professor
London School of Hygiene and Tropical Medicine, London, UK

Elena Pizzo - Principal Research Fellow
Department of Applied Health Research, University College London, London, UK

Fiona Aspinal - Senior Research Associate
Department of Applied Health Research, University College London, London, UK

Sarah Jasim - Senior Research Associate
Care Policy Evaluation Centre, London School of Economics & Political Science, London, UK

Rafael Gafoor - Senior Research Associate
Department of Applied Health Research, University College London, London, UK

Jean Ledger - Senior Research Associate
Department of Applied Health Research, University College London, London, UK

Raj Mehta – Patient Advisor
Department of Applied Health Research, University College London, London, UK

James Mason - Professor of Health Economics
Warwick Medical School, University of Warwick, Coventry, UK

Peter Martin - Lecturer in Applied Statistics
Department of Applied Health Research, University College London, London, UK

Naomi J. Fulop - Professor of Health Care Organisation and Management
Department of Applied Health Research, University College London, London, UK

Steve Morris - RAND Professor of Health Services Research
Institute of Public Health, University of Cambridge, Cambridge, UK

Rosalind Raine - Professor of Health Care Evaluation
Department of Applied Health Research, University College London, London, UK

*Joint first author

Correspondence to
Dr Helen Barratt, Department of Applied Health Research, University College London, London WC1E 6BT, UK.
h.barratt@ucl.ac.uk

For peer review only

Abstract

Objective: To evaluate the impact of the 'Getting it Right First Time' (GIRFT) national improvement programme in orthopaedics, which started in 2012.

Design: Mixed-methods study comprising statistical analysis of linked national datasets (National Joint Registry; Hospital Episode Statistics; Patient Reported Outcomes); economic analysis; and qualitative case studies in six NHS Trusts.

Setting: NHS elective orthopaedic surgery in England.

Participants: 736,088 patients who underwent primary hip or knee replacement at 126 NHS Trusts between 1 April 2009-31 March 2018, plus 50 NHS staff.

Intervention: Improvement bundle including 'deep dive' visits by senior clinician to NHS Trusts, informed by bespoke set of routine performance data, to discuss how improvements could be made locally.

Main outcome measures: Number of procedures conducted by low volume surgeons; use of uncemented hip implants in patients >65; arthroscopy in year prior to knee replacement; hospital length of stay; emergency readmissions within 30 days; revision surgery within one year; health-related quality of life; and functional status.

Results: National trends demonstrated substantial improvements beginning prior to GIRFT. Between 2012-2018, there were reductions in procedures by low volume surgeons (ORs (95% CI) hips 0.58 (0.53 to 0.63), knees 0.77 (0.72 to 0.83)); uncemented hip prostheses in >65s (OR 0.56 (0.51 to 0.61)); knee arthroscopies before surgery (OR 0.48 (0.41 to 0.56)); and mean length of stay (hips -0.90 (-1.00 to -0.81), knees -0.74 days (-0.82 to -0.66)). The additional impact of visits was mixed and comprised an overall economic saving of £431,848 between 2012-2018, but this was offset by the costs of the visits. Staff reported that GIRFT's influence ranged from procurement changes to improved regional collaboration.

Conclusion: Nationally, we found substantial improvements in care, but the specific contribution of GIRFT cannot be reliably estimated due to other concurrent initiatives. Our approach enabled additional analysis of the discrete impact of GIRFT visits.

For peer review only

Strengths and limitations of this study

- We report the first, independent evaluation of the high profile Getting It Right First Time NHS improvement programme.
- Our mixed method approach enabled us to provide a comprehensive and robust understanding of GIRFT, exploring the impact of the programme from different perspectives.
- Our linked dataset allowed us to examine a range of measures, as well as estimating the specific contribution of Trust visits, whilst the case study analysis provided further insights from the perspective of Trust staff.
- We could not examine some key GIRFT target measures (e.g. procurement, litigation rates) because appropriate data were not available.

For peer review only - http://bmjopen.bmj.com/site/about/guidelines.xhtml

- We also could not capture costs incurred by Trusts, because activities were not consistently tracked.

Introduction

'Getting it Right First Time' (GIRFT) is one of the largest improvement programmes in the NHS. It began in orthopaedics in 2012 with the publication of the first GIRFT report, recommending changes to improve outcomes and reduce costs.[1] Following government investment totalling £62.8m, GIRFT now operates in 44 different specialities, or clinical workstreams.[2] GIRFT is an improvement 'bundle' - a small number of interventions performed together to improve care.[3] Clinical leadership is fundamental: GIRFT was established by a senior surgeon, who leads the orthopaedic workstream and now chairs the wider programme. Each workstream is chaired by a clinical lead from the relevant speciality. The programme includes components operating at local (i.e. NHS provider Trusts) and national (i.e. across England) levels. Local components include 'deep dive' visits to Trusts, whilst national components include national reports describing how unwarranted variations can be addressed. Initial first visits were piloted at a small number of Trusts in 2013, then replicated across the country in orthopaedics and other workstreams from 2015. Before each visit, Trusts are sent a bespoke 'datapack,' collated by GIRFT, describing their performance on over 100 variables, drawn from sources including Hospital Episode Statistics (HES) and national audits, such as the National Joint Registry (NJR). Data include use of evidence-based procedures and costs. Datapacks bring data sources together and make them accessible, facilitating comparison to national and peer group averages. Discussion at the meeting is driven by the datapack and tailored to the Trust. Attendees, comprising clinicians, managers and other relevant professionals, identify where and how improvements could be made. Revisits follow a similar format with Trusts reporting changes made since the first visit.

There are few examples of initiatives on this scale, and consequently few evaluations. The American College of Surgeons National Surgical Quality Improvement Program (NSQIP) provides data to drive improvement. However, only around 10% of US hospitals participate.[4] In the UK, the national Perioperative Quality Improvement Programme (PQIP) will collate and feedback data about surgical complications.[5] NHS Trusts are also subject to inspections by the Care Quality Commission, whilst some National Clinical Audits also provide written feedback.[6] However, GIRFT clinical leads visit all Trusts to discuss their datapack, as well as opportunities for improvement. GIRFT reflects evidence that measurement alone is insufficient.[7] The effectiveness of feedback is dependent on hard' and 'soft'[8] incentives, including using data to support change and holding participants to account at revisits.[9] However, although GIRFT visits Trusts twice, sometimes more, the largest improvements in NSQIP were seen where providers had participated for many years.[10] Improvements were also more marked at sites with pre-existing internal improvement programmes.[11] GIRFT makes use of system (top-down) leadership, but designated local (bottom-up) leadership within Trusts may also be important.[12] Despite the limited evidence base, GIRFT has been expanded across the NHS, in the absence of independent evaluation.

We therefore evaluated the effectiveness and cost-effectiveness of the GIRFT orthopaedic workstream, focussing on the most common elective procedures, primary hip and knee replacement.[13] The few evaluations of national programmes focus mainly on quantitative process and outcome measures, and hence may miss wider effects. Consequently, we undertook a multicomponent analysis. The first described national trends in processes and outcomes over time, starting before GIRFT. However, this cannot disentangle the relative

For peer review only - http://bmjopen.bmj.com/site/about/guidelines.xhtml

impact of GIRFT from other concurrent national initiatives that targeted similar measures. For example, Lord Carter's review of NHS hospital efficiency took place between June 2014 and February 2016, and made recommendations about tackling 'unwarranted variation' in key resource areas such as staffing, diagnostics and procurement. NHS Right Care originated as part of the Quality, Innovation, Productivity and Prevention programme in 2009 and is still ongoing. It supports local economies to improve population health care and address performance variations. Our second analysis focused on a specific component of GIRFT, first visits to Trusts, to elucidate their *additional* local impact over and above the national trends. We also undertook an economic analysis to evaluate the cost impacts of visits, and a qualitative exploration of the impact of GIRFT from the perspective of Trust staff.

Methods

Design

We employed a mixed methods approach, using both quantitative and qualitative data to consider the various impacts of GIRFT from different perspectives and at different levels (national and Trust). In the quantitative analysis, we examined eight key GIRFT target measures. We first describe changes in these at national level. We then exploit variation in the timing of GIRFT visits to assess the *additional* impact of initiating local involvement *after allowing for national trends*. We also undertook an economic analysis to assess costs and savings attributable to the visits. Finally, we used qualitative methods to explore how staff perceived that GIRFT had impacted practice at six case study sites.

Quantitative methods

Quantitative data sources

We used linked data from NJR, HES and Patient Reported Outcomes (PROMs) to identify patients aged 18 or over who underwent an elective primary hip or knee replacement between 1 April 2009-31 March 2018, at 126 English Trusts that received a first GIRFT visit by November 2017. We excluded eight Trusts visited later because they would contribute no post-visit data to the analysis. We included primary procedures eligible for the PROMs programme.[14] Metal-on-metal implants were excluded because they were subject to specific regulatory measures.[15]

Variables

We identified eight measures that were GIRFT priorities and available from the dataset. We were unable to use data on some priority indicators, such as litigation and procurement, because they are not publicly available. Process measures were: (i) procedures conducted by low volume surgeons (=<35 similar procedures or =< 10 unicondylar/ patella-femoral knee replacements per year); (ii) use of uncemented hip implants in patients >65; (iii) arthroscopy in year prior to knee replacement. GIRFT sought to reduce these, citing evidence that higher surgeon volumes equate to better outcomes; knee arthroscopy is not effective; and uncemented hip implants in older patients have higher revision rates.[1] There is no commonly accepted threshold for surgeon volume. We therefore used the thresholds =<35 similar procedures or =< 10 unicondylar/ patella-femoral knee replacements per year based on clinical input, the literature on 'low volume' surgical thresholds in orthopaedic surgery, discussion with the GIRFT programme team and recommendations in their 2015 programme report.[2] Outcome measures comprised: (i) hospital length of stay for index procedure; (ii) emergency readmissions within 30 days; (iii) revision surgery within one year; and in the

For peer review only - http://bmjopen.bmj.com/site/about/guidelines.xhtml

subset of patients who participated in the national PROMs programme (iv) health-related quality of life (EQ-5D)[16] and (v) functional status (Oxford Hip/ Knee Score (OHKS)).[17] GIRFT sought to minimise the first three to reduce associated costs. We included EQ5D and OHKS to facilitate economic evaluation. We identified revisions from NJR and HES,[18] measuring patient-reported outcomes at six months.[14] We adjusted for: age in years; ethnicity (white, not white, or missing/not reported); sex; quintile of Index of Multiple Deprivation; Charlson comorbidity[19] (HES); and American Society of Anaesthesiologists grade (NJR).

Statistical analyses

We examined proportions and means of measures before GIRFT started (2009/10-2011/12), and in the final two years of the study (2016/17-2017/18). In our analysis of national trends, we also estimated year-on-year changes in comparison with 2012/13, the year GIRFT began, using casemix-adjusted hierarchical logistic and linear regression.

We analysed the additional impact of initiating visits using a pre-post design. First visit dates defined pre- and post- periods for Trusts. The first visit marked the start of GIRFT's local involvement with an individual Trust. Prior to this, a Trust would be aware of GIRFT's national work, but would not have received the intervention tailored to their individual circumstances. At the start of the programme, the GIRFT team contacted all eligible Trusts, and the timetable of the first visits was based on the order in which sites responded, rather than, for example, orthopaedic performance data. Nevertheless, the order of visits was not random, so we divided Trusts into early, middle and late groups in a 1:2:1 ratio, to examine whether changes differed by visit timing. The ratio was specified *a priori* to split Trusts by lower and upper quartiles, ensuring a minimum of 30 per group. We used hierarchical logistic and linear regression models adjusted for casemix variables with clustering at Trust-level to estimate changes in levels for each measure. We controlled for temporal trends using fractional polynomials, and pre-operative scores in PROMs analyses. The impact in early, middle and late groups were estimated using interactions between group and the change in levels. The only casemix variables with missing data were ethnicity (3.8% and 2.7% of THR and TKR patients respectively) and Index of Multiple Deprivation (Table 1). We used a complete case analysis but included a missing category for ethnicity. Cases with a missing deprivation score represented only 0.3% of the analysis sample and were excluded from the complete case analysis. We allowed for implementation delays by excluding procedures in the three months after each initial Trust visit, based on a GIRFT case study indicating that improvements occured within three months.[20] We used a longer 15-month exclusion when analysing arthroscopies, because these were measured in the 12 months before surgery and hence might overlap the visit date.

Economic analysis methods

Conventionally, economic evaluation compares costs and outcomes of an intervention and comparator. However, it was not possible to use a comparator because GIRFT visited Trusts at different times. Instead, we compared the impact of visits at Trust level with expected costs in the absence of GIRFT. We examine: (1) cost of the visits; (2) costs incurred by Trusts to implement recommendations; and (3) costs or savings resulting from the visits in the limited measures publicly available for analysis. (Figure 1)

Information from the GIRFT programme team enabled us to calculate visit costs at Trust-level. We collected data on costs incurred by Trusts via a national survey, distributed electronically on our behalf, in early 2018, by the programme team, to GIRFT 'champions' in each Trust. It included questions about five GIRFT recommendations that could have an economic impact: implementation of ring-fenced beds; introduction of extended physiotherapy services;

For peer review only - http://bmjopen.bmj.com/site/about/guidelines.xhtml

changes in use of theatre loan kits; reductions in activity by low volume surgeons; and improvements to theatre efficiency.

We assessed the economic impact of changes using the results of the Trust-level analysis above, quantified using NHS cost data.[21] We used the marginal effects estimated in the statistical models to estimate the economic impact of the visits where a statistically significant change was observed.

Qualitative methods

As outlined in the study protocol,[13] our evaluation included several qualitative elements, including interviews with the GIRFT programme team and national health leaders (to understand the development of GIRFT and its evolution over time), as well as focus groups with patients (to understand their views about the content of the GIRFT programme). As these elements did directly contribute to our assessment of the impact of GIRFT, their findings will be reported elsewhere. We used a case study approach to explore the implementation of GIRFT in individual NHS Trusts, including staff perceptions about whether and how GIRFT impacted practice locally.[22]

Case study sites

We purposively sampled six Trusts in England, representing a spread of hospital types (district general and teaching hospitals) and setting (region and rural versus urban).

Qualitative data collection

We have described our data collection methods previously.[13] Here, we report data collected via semi-structured interviews with staff at the six case study sites, between Oct 2016-May 2019 (Appendix 1). The interview topic guide was developed with input from the evaluation team (e.g. to incorporate questions about resource costs and implementation) and informed by scoping discussions conducted with the team delivering GIRFT, to understand the programme components (see Appendix 2). It was piloted prior to the interviews, and then refined iteratively as the study progressed, to take account of emerging findings. Interviewees included surgeons and other staff present at the first GIRFT visit or knowledgeable about local improvement. Interviews lasted between 20-60 minutes and were audio-recorded for full transcription.

Qualitative data analysis

We analysed data thematically, within and across cases, combining inductive and deductive (informed by the aims of the GIRFT programme) approaches.[23]

Patient and Public Involvement

From the inception of the study, we worked with the NIHR CLAHRC North Thames lay Research Advisory Panel to to refine the protocol and ensure that the proposed research appropriately reflected the priorities, experience, and preferences of patients. Through this, we identified a patient representative (RM) who agreed to join the study steering board. He has subsequently played a collaborative role in refining the research design, interpreting findings and disseminating results. The research reported here did not directly involve patients, so RM did not play a role in recruitment to the study.

For peer review only - http://bmjopen.bmj.com/site/about/guidelines.xhtml

Ethics approval

Granted by NHS North West - Liverpool East Research Ethics Committee (16/NW/0654).

Results

Quantitative findings

Study population

337,279 patients who underwent a hip replacement and 398,809 who received a knee replacement in 126 NHS Trusts, between 1 April 2009-31 March 2018. (Figures 2+3) See Table 1 for patient characteristics.

1) National trends

Process measures

Nationally, there were substantial improvements in process measures, often beginning before 2012 when GIRFT (Figures 4+5). Comparing 2009-2012 and 2015-2018, reductions varied from 29% fewer uncemented hips to a 58% reduction in knee arthroscopy. (Table 2) Reductions in procedures by low volume surgeons began prior to GIRFT for hips and knees. Odds ratios for 2017/18 versus 2012/13 were 0.58 (95% CI 0.53, 0.63) for hips and 0.77 (95% CI 0.72, 0.83) for knees. Reductions in uncemented hips and knee arthroscopy also began prior to 2012, but the largest occurred as GIRFT progressed with odds ratios for 2017/18 versus 2012/13 of 0.56 (95% CI 0.51, 0.61) and 0.48 (95% CI 0.41, 0.56) respectively.

Outcome measures

Mean length of stay reduced by just over one day for both hip and knee patients between 2009-2012 and 2015-2018. The largest reductions began before GIRFT (Figures 4+5). Mean differences between 2012/13 and 2017/18 were -0.90 days (95% CI -1.00, -0.81) for hips and -0.77 days (95% CI -0.82, -0.66) for knees. There was some evidence that one-year knee revisions declined by 2017/18, but little evidence of a change in hips. Post-operative quality of life and functional status improved, but there was little change in emergency readmissions (Table 2 and Figures 4+5).

2) Additional effect of initiating 'deep dive' involvement at individual Trusts

GIRFT's first visits to Trusts occurred between September 2013 and November 2017 with half occurring between February and July 2014. (Table 3)

Process measures

The additional effects of the visits on process measures, after controlling for national trends, differed between the earliest Trusts visited and the middle and later groups. In the earliest group, we observed reductions in procedures by low volume surgeons. Conversely, this increased in the middle and late groups, but use of uncemented hips reduced. (Table 3)

For peer review only - http://bmjopen.bmj.com/site/about/guidelines.xhtml

Outcome measures:

Following visits, mean length of stay increased for the middle and late groups, but decreased in hip patients in the earlier group. However, estimated effects were small in comparison with national trends. We found limited evidence of an impact on other outcomes. (Table 3).

Economic analysis

The estimated cost to deliver the Trust visits was £491,420 (Component 1). This is a 'sunk' cost, including £10,769 transport costs (total return trip mileage); £150,000 for datapacks; £111,916 GIRFT staff time; and £106,820 opportunity cost of Trust staff time attending the meetings.

Our national survey provided mixed reports about the implementation of recommendations so it was not possible to accurately estimate costs. Therefore, although Trusts incurred costs, these could not be included (Component 2).

Although length of stay increased after the visits ($p<0.05$), this was not included in our economic analysis. Trusts receive the same payment for each inpatient stay, up to the tariff 'trim point' (currently nine days). However, average length of stay remained below this, despite the observed increase, so there was no extra monetary cost. Nevertheless, increased length of stay does come with an opportunity cost, as the bed is not available for another patient. As there was no change in EQ-5D, used to calculate quality adjusted life years, we were unable to undertake a cost-effectiveness analysis. Instead, we conducted a cost-consequences analysis. We observed positive and negative changes after GIRFT visits (Table 3) and additional costs partly outweighed savings (Table 4). The overall impact of the statistically significant changes, for the limited number of measurable variables, not including length of stay, was a saving of £431,848 (Component 3).

Case-study analysis

In this paper, our goal is to evaluate the effectiveness and cost-effectiveness of the GIRFT programme, focussing on processes and outcomes of care. We therefore restrict our reporting here to the qualitative data sources where the impact of GIRFT on patient care was explored: interviews conducted at our six case study sites. We conducted 50 interviews across six sites (Appendix 1). Interviewees described five types of impact, operating at three levels: individual, Trust and regional (Figure 6). These ranged from changes to implant selection, to improved networking within Trusts and across regions. However, GIRFT particularly impacted ways of working at Trust-level, for example, catalysing planned improvements.

Interaction within and between the three levels of impact was key. For example, reducing low volume surgery depended on regional partners forming networks. Similarly, increasing the number of procedures conducted per day required within-organisation negotiation to access theatre time. Visits were most successful when GIRFT aligned with Trust priorities (e.g. rationalising procurement - Site 3). They were less effective where GIRFT measures were not a local priority because of more immediate demands, for example, financial pressures

A range of factors, in addition to GIRFT, impacted practice. These included the concurrent roll out of other initiatives. For example, the Carter Review impacted procurement (Sites 2 and 6), whilst reductions in arthroscopies were driven by the Payment by Results Assurance Framework (Site 2).

For peer review only - http://bmjopen.bmj.com/site/about/guidelines.xhtml

Discussion

This is the first independent evaluation of GIRFT. We found substantial improvements in orthopaedic care during the first six years of the programme, notably reductions in uncemented hip prostheses, knee arthroscopies and length of stay. However, these started before GIRFT. It was not possible to estimate the distinct contribution of the programme, because of other concurrent initiatives with common goals (e.g. Carter Review). We also estimated the additional impact of GIRFT visits. We found a mix of positive and negative effects, generally small compared to overall improvements and differing between the earliest and latest Trusts visited. It is important to note, though, that the eight measures we analysed in our quantitative analyses, and targeted by GIRFT, relate to direct patient care. Staff at our case study sites reported that the programme had had an impact, but the effects that they described related much more to ways of working at Trust-level (e.g. improved networking) rather than direct patient care.

Our mixed method approach has enabled us to provide a comprehensive and robust understanding of GIRFT, using quantitative and qualitative data to explore the impact of the programme from different perspectives. Previous similar evaluations tend to focus on quantitative analyses. Our comprehensive linked dataset allowed us to examine a range of measures, as well as estimating the specific contribution of Trust visits. The only variables with missing data were ethnicity (3.8% and 2.7% of THR and TKR patients respectively), for which we included a 'missing' category, and Index of Multiple Deprivation. We excluded the latter from the complete case analysis, but as this represented only 0.3% of the analysis sample, it is unlikely to have had a major impact on our findings. The case study analysis provided further insights from the perspective of Trust staff. However, we measured changes at three months post-visit, and although some made improvements within this window, it may not have been sufficient for others. A further limitation is that we could not examine other key target measures. Procurement data were only available for 2017-19 and could not be compared to previous years. Although litigation data are available, our previous work[24] demonstrates a significant lag from incident to resolution. Consequently, it would not be possible to determine whether changes were an impact of GIRFT or other policies (e.g. Sign up to Safety). Other outcomes, such as 5- and 10-year revisions, were beyond our time frame, as were impacts after 2018. We also could not capture costs incurred by Trusts, because activities were not consistently tracked. The increased net cost associated with the programme is therefore a conservative estimate. Finally, our qualitative interviews took place several months after the first GIRFT visits, creating a risk of recall bias.[25]

In 2020, the GIRFT team published an internal evaluation[20] describing how the orthopaedic workstream had supported Trusts to release 'financial opportunities' of £696million. Our findings differ for a number of reasons. First the internal evaluation was limited to descriptive analyses of national trends between 2013/14 and 2018/19. This is broadly consistent with our national trend analysis, although we adjusted for casemix and included data from 2009 to explore changes prior to GIRFT. In contrast, the GIRFT team attributed all trend changes to the programme. Second, they limited their economic analysis to the impact on processes and outcomes, whereas we also examined the cost of the visits and costs incurred by Trusts. Finally, our diverse case study sites facilitated cross-case comparison, to create a detailed contextual picture. The internal evaluation includes individual case studies which exemplify success. Early narrative reviews of GIRFT were published by the Kings Fund[26] and NHS Providers[27] However, these are not formal evaluations.

For peer review only - http://bmjopen.bmj.com/site/about/guidelines.xhtml

Our finding of improvements in processes but less clear changes in outcomes is consistent with evidence that improvement initiatives generally have greater impact on processes of care than patient outcomes.[28] Improvements observed before 2012 may be explained by GIRFT identifying existing best practice to share more widely. The additional impact of visits was mixed, with no consistent pattern across the cohorts. In some cases, performance worsened immediately afterwards. There may have been underlying differences between Trusts visited earlier and later, but visits were just one part of the programme and we are aware that other components of GIRFT, such as national reports, had impacted the care provided at case study sites. One further possible explanation is that later cohorts had made changes prior to their visit because of information they gleaned from Trusts visited earlier in the process. Although some of the Trusts were familiar with the overall recommendations being made by GIRFT, this could equally have been because they were also priorities for other national programmes being rolled out at the same time. As data collection at the case study sites took place before the quantitative analyses, and therefore we did not know about the variations in individual process and outcome measures at the time the interviews took place, participants were not directly asked about them. It would also have been a challenge to draw firm conclusions about the causes of these differences from just six sites. However, our findings reflect other literature illustrating the challenges for improvement programmes in outperforming the secular trend, including the possibility that the programmes themselves may be implicated in that trend.[29]

GIRFT is one of the largest improvement initiatives in the NHS. Our analysis demonstrates significant improvements in orthopaedic care, which began prior to GIRFT. Changes observed over the past 10 years are likely attributable to both GIRFT and other concurrent initiatives, but we cannot determine the relative contributions of these. The additional impact of visits was mixed. Given the substantial cost and expansion of the programme, ongoing monitoring and access to additional relevant data, including details of Trust activities, as well as early engagement with rigorous evaluation design (e.g. stepped wedge approaches), are recommended to enhance the ability to hold GIRFT and other national improvement progammes to account.

For peer review only

Tables

Table 1: Characteristics of patient population*

	Hip replacement patients (n = 337,279)		Knee replacement patients (n = 398,809)	
	**N**	**%**	**N**	**%**
**Age**				
10 – 19	37	0.0%	-	-
20 – 29	1,553	0.5%	140	0.0%
30 – 39	4,805	1.4%	851	0.2%
40 – 49	16,765	5.0%	10,443	2.6%
50 – 59	46,753	13.9%	53,736	13.5%
60 – 69	96,371	28.6%	131,197	32.9%
70 – 79	115,840	34.4%	144,271	36.2%
80 – 89	51,900	15.4%	55,873	14.0%
90 and over	3,255	1.0%	2,298	0.6%
**Sex**				
Male	136,468	40.5%	169,959	42.6%
Female	200,811	59.5%	228,850	57.4%
**Ethnicity**				
White	317,671	94.4%	360,742	90.5%
Other	6,941	2.2%	27,327	6.9%
Missing	12,667	3.8%	10,740	2.7%
**Index of multiple deprivation quintile**				
Least Deprived	78,059	23.1%	84,401	21.2%
Less Deprived	80,505	23.9%	88,714	22.2%
Deprived	61,831	18.3%	77,281	19.4%
More Deprived	59,340	17.6%	75,173	18.9%
Most Deprived	56,520	16.8%	72,153	18.1%
Missing*	1,024	0.3%	1,087	0.3%
**Charlson comorbidity score**				
0	210,385	62.4%	232,953	58.4%
>= 1	126,894	37.6%	165,856	41.6%
**American Society of Anaesthesiologists Score**				
Healthy	40,379	12.0%	32,329	8.1%
Mild Systemic	227,471	67.4%	284,064	71.2%
Severe/Moribund	69,429	20.6%	82,416	20.7%

[†]IMD (Index of Multiple Deprivation) |[‡] ASA (American Society of Anaesthesiologists Score) | [¥]OHKS Oxford Hip Knee Score |
*Cases missing an IMD score were excluded from the complete case analysis

For peer review only - http://bmjopen.bmj.com/site/about/guidelines.xhtml

Table 2: Unadjusted process and outcome measures across the study population at the beginning and end of the study period, 1 Apr 2009-31 Mar 2018

		Beginning of study period (01/04/09-31/3/12)	End of study period (01/04/16-31/03/18)
**Process measures**			
Low volume surgery (<35 per annum) (%)	Hip	15.9% (95% CI 15.70 to 16.2)	9.1% (95% CI 8.9 to 9.3)
	Knee	18.6% (95% CI 18.4 to 18.8)	13.4% (95% 13.2 to 13.7)
Uncemented hip implant in >65s (%)		31.7% (95% CI 31.4 to 32.1)	22.6% (95% CI 22.20 to 23.0)
Arthroscopy in previous 12m (%)		6.5% (95% CI 6.3 to 6.56)	3.4% (95% CI 3.3 to 3.5)
**Outcome measures**			
Length of stay (median and mean days)	Hip	5 (IQR 3 to 6) 5.6 (95% CI 5.6 to 5.6)	3 (IQR 2 to 5) 4.3 (95% CI 4.3 to 4.4)
	Knee	5 (IQR 3 to 6) 5.5 (95% CI 5.5 to 5.5)	4 (IQR 3 to 5) 4.3 (95% CI 4.3 to 4.4)
Readmissions in 30 Days (%)	Hip	4.7% (95% CI 4.5 to 4.8)	4.6% (95% CI 4.4 to 4.7)
	Knee	5.2% (95% CI 5.1 to 5.3)	5.0% (95% CI 4.9 to 5.2)
Revisions in 12 Months (%)	Hip	0.77% (95% CI 0.72 to 0.82)	0.63% (95% CI 0.58 to 0.69)
	Knee	0.44% (95% CI 0.40 to 0.48)	0.37% (95% CI 0.33 to 0.41)
Post-operative Oxford Hip/ Knee Score (mean)	Hip	38.1 (95% CI 38.0 to 38.2)	39.2 (95% CI 39.2 to 39.3)
	Knee	33.7 (95% CI 33.6 to 33.8)	35.3 (95% CI 35.2 to 35.4)
Post-operative EQ5D score (mean)	Hip	0.76 (95% CI 0.76 to 0.77)	0.78 (95% CI 0.78 to 0.78)
	Knee	0.70 (95% CI 0.70 to 0.70)	0.73 (95% CI 0.73 to 0.73)

For peer review only - http://bmjopen.bmj.com/site/about/guidelines.xhtml

Table 3: Changes in process and outcome measures after first GIRFT visit by Trust cohort*
(Statistically significant values in bold)

		Early cohort 10/09/13-02/02/14 (n=31 Trusts)	Middle cohort 03/02/14-06/07/14 (n=61 Trusts)	Late cohort 07/07/14-27/11/17 (n=34 Trusts)
**Process measures** (ORs below 1 indicate improvement)				
Low volume surgery (<35 per annum) (OR)	Hip	**0.92 (95% CI 0.87 to 0.98)**	**1.12 (95% CI 1.06 to 1.18)**	**1.11 (95% CI 1.04 to 1.19)**
	Knee	**0.92 (95% CI 0.87 to 0.97)**	**1.13 (95% CI 1.08 to 1.19)**	**1.19 (95% CI 1.13 to 1.26)**
Uncemented hip implant in >65s (OR)		1.07 (95% CI 1.00 to 1.16)	**0.82 (95% CI 0.77 to 0.87)**	**0.73 (95% CI 0.69 to 0.78)**
Arthroscopy in previous 12m (OR)		1.09 (95% CI 0.99 to 1.20)	1.08 (95% CI 0.99 to 1.18)	**1.19 (95% CI 1.06 to 1.33)**
**Outcome measures** (Negative change in length of stay, ORs below 1, and positive changes in Oxford Hip/Knee and EQ5D scores indicate improvement)				
Length of stay (change in mean days)	Hip	**-0.14 (95% CI -0.20 to -0.07)**	**0.09 (95% CI 0.03 to 0.14)**	**0.11 (95%CI 0.04 to 0.18)**
	Knee	-0.01 (95% CI -0.06 to 0.04)	**0.07 (95% CI 0.03 to 0.12)**	**0.06 (95% CI 0.01 to 0.12)**
Readmissions within 30 days (OR)	Hip	1.01 (95% CI 0.92 to 1.10)	1.07 (95% CI 0.99 to 1.16)	1.03 (95% CI 0.94 to 1.13)
	Knee	1.08 (95% CI 0.99 to 1.17)	1.03 95% CI (0.96 to 1.11)	**1.16 (95% CI 1.06 to 1.26)**
Revisions within 12 months (OR)	Hip	1.14 (95%CI 0.91 to 1.44)	1.06 (95% 0.87 to 1.30)	1.16 (95% CI 0.92 to 1.47)
	Knee	1.14 (95% CI 0.94 to 1.38)	0.95 (95% 0.82 to 1.10)	0.84 (95% CI 0.68 to 1.04)
Oxford Hip/ Knee Score (change in mean score)	Hip	**-0.12 (95% CI -0.33 to -0.09)**	-0.17 (95% CI -0.35 to 0.01)	-0.27 (95% CI -0.48 to 0.05)
	Knee	**-0.37 (95% CI -0.58 to -0.15)**	**-0.34 (95% CI -0.53 to -0.16)**	**-0.37 (95% CI -0.59 to -0.14)**
EQ-5D (change in mean score)	Hip	0.00 (95% CI 0.00 to 0.01)	0.00 (95% CI 0.00 to 0.01)	0.00 (95% CI 0.00 to 0.00)
	Knee	0.00 (95% CI-0.01 to 0.01)	0.00 (95% CI-0.01 to 0.01)	-0.01 (95% CI-0.01 to 0.00)

*Odds Ratios and linear regression coefficients for post-first GIRFT visit (reference pre-first GIRFT visit) adjusted for patient baseline characteristics and time variables

For peer review only - http://bmjopen.bmj.com/site/about/guidelines.xhtml

Table 4: Summary of GIRFT economic impact following first GIRFT visit by Trust cohort (2019/2020 GBP)
(Positive values are costs; negative values are savings; statistically significant values are in bold, with reference to pre-first GIRFT visit.)

		Early cohort 10/09/13-02/02/14 (n=31 Trusts)	Middle cohort 03/02/14-06/07/14 (n=61 Trusts)	Late cohort 07/07/14-27/11/17 (n=34 Trusts)	Total results for all cohorts
**Process measures**					
Uncemented hip implant in >65s		87,654	**-444,107****	**-357,151****	- 713,604
Arthroscopy in previous 12m		242,365	330,927	**320,467****	893,759
**Outcome measures**					
Length of stay	Hip	-198,320	**5,641,544****	**2,513,717****	7,956,941
	Knee	**1,048,798****	**5,555,429****	**2,333,021****	8,937,249
Readmissions within 30 days	Hip	3,332	37,799	7,364	48,495
	Knee	37,325	21,785	**48,943****	108,053
Revisions within 12 months	Hip	411,777	290,035	327,938	1,029,750
	Knee	305,944	-182,231	-238,211	- 114,498
**Totals**					
**Overall economic impact[a]**		**1,938,876**	**11,251,181**	**4,956,088**	**18,146,144**
Savings [a]		-198,320	-626,338	-595,362	- 1,420,020
Incremental cost [a]		2,137,195	11,877,519	5,551,450	19,566,164
**Statistically significant economic impact [b]**		1,048,798	10,752,866	4,858,997	16,660,661
**Statistically significant economic impact (excluding LOS)[b]**		-	**-444,107****	**12,259****	**- 431,848****

[a] This is the sum of economic impact (saving or incremental cost) irrespective of statistically significant results.
[b] This is the sum of the economic impact taking into account only statistically significant results. ** statistically significant results at the 5% level

For peer review only - http://bmjopen.bmj.com/site/about/guidelines.xhtml

Figure legends

Figure 1: The economic components of GIRFT orthopaedic evaluation

Figure 2: Study population – total hip replacement patients
Patient Reported Outcomes Measures (PROMS) data collected from April 2009 for consenting patients only

Figure 3: Study population – total knee replacement patients
Patient Reported Outcomes Measures (PROMS) data collected from April 2009 for consenting patients only

Figure 4: Trends in process and outcome measures for primary hip replacements (2009/10-2017/18)

Figure 5: Trends in process and outcome measures for primary knee replacements (2009/10-2017/18)

Figure 6: Summary of impacts attributed to GIRFT orthopaedic visits at six case-study site

For peer review only

For peer review only

For peer review only - http://bmjopen.bmj.com/site/about/guidelines.xhtml

Contributors
HB was principal investigator. HB and RR initiated the research. HB, AH, EP, NJF, SM and RR designed the evaluation. SJ, AH and RG created the linked dataset. RG and AH conducted the quantitative analyses; PM provided statistical advice. SJ and EP designed the Trust survey and analysed the resultant data. EP and SM led the economic analysis. SJ and JL were responsible for qualitative data collection; FA, JL, SJ, HB and NJF analysed the qualitative data. HB, AH, EP, FA and RR drafted the manuscript and SJ, RG, JL, RM, JM, PM, NJF and SM contributed to reviewing and revision. HB, AH, EP, FA, SJ, RG, JL, RM, JM, PM, NJF, SM and RR approved the final version. HB, AH, EP, FA, SJ, RG, JL, RM, JM, PM, NJF, SM and RR had full access to all the data in the study and accept responsibility to submit for publication. HB will act as guarantor.

Declaration of interests
All authors have completed the ICMJE uniform disclosure form at www.icmje.org/coi_disclosure.pdf and declare: HB, AF, EP, FA, SJ, RG, JL, and RR are funded in full or in part by the National Institute for Health Research ARC North Thames. RR and NJF are NIHR Senior Investigators; no financial relationships with any organisations that might have an interest in the submitted work in the previous three years; no other relationships or activities that could appear to have influenced the submitted work. The views expressed in this publication are those of the authors and not necessarily those of the National Institute for Health Research or the Department of Health and Social Care.

Transparency declaration
The corresponding author (the manuscript's guarantor) affirms that the manuscript is an honest, accurate, and transparent account of the study being reported; that no important aspects of the study have been omitted; and that any discrepancies from the study as originally planned have been explained.

Funder
This report is independent research funded by the National Institute for Health Research ARC North Thames (award/ grant number is not applicable). The views expressed in this publication are those of the authors and not necessarily those of the National Institute for Health Research or the Department of Health and Social Care.

Role of the funder
The funder had no role in study design, data collection, data analysis, data interpretation, or writing of the report.

Data sharing
In line with the data sharing agreements between University College London and NHS Digital/ HQIP, aggregate small number suppressed outputs for the study period (1 April 2009-31 March 2018) are available on request from the corresponding author, Dr Helen Barratt (h.barratt@ucl.ac.uk).

Licence statement
I, the Submitting Author has the right to grant and does grant on behalf of all authors of the Work (as defined in the below author licence), an exclusive licence and/or a non-exclusive licence for contributions from authors who are: i) UK Crown employees; ii) where BMJ has agreed a CC-BY licence shall apply, and/or iii) in accordance with the terms applicable for US Federal Government officers or employees acting as part of their official duties; on a worldwide, perpetual, irrevocable, royalty-free basis to BMJ Publishing Group Ltd ("BMJ") its licensees and where the relevant Journal is co-owned by BMJ to the co-owners of the Journal, to publish the Work in BMJ Open and any other BMJ products and to exploit all rights, as set out in our licence.

For peer review only - http://bmjopen.bmj.com/site/about/guidelines.xhtml

The Submitting Author accepts and understands that any supply made under these terms is made by BMJ to the Submitting Author unless you are acting as an employee on behalf of your employer or a postgraduate student of an affiliated institution which is paying any applicable article publishing charge ("APC") for Open Access articles. Where the Submitting Author wishes to make the Work available on an Open Access basis (and intends to pay the relevant APC), the terms of reuse of such Open Access shall be governed by a Creative Commons licence – details of these licences and which Creative Commons licence will apply to this Work are set out in our licence referred to above.

Acknowledgements
We thank Patricia Hallam for administrative support for the study. We thank the patients and staff of all the hospitals in England, Wales and Northern Ireland who have contributed data to the National Joint Registry. We are grateful to the Healthcare Quality Improvement Partnership (HQIP), the NJR Research Committee and staff at the NJR Centre for facilitating this work. The authors have conformed to the NJR's standard protocol for data access and publication. The views expressed represent those of the authors and do not necessarily reflect those of the National Joint Registry Steering Committee or the Healthcare Quality Improvement Partnership (HQIP) who do not vouch for how the information is presented.

For peer review only

References

1. Briggs T. *Getting it Right First Time: improving the quality of orthopaedic care within the National Health Service in England*. London: British Orthopaedic Association; 2012.

2. Briggs T. *Getting it Right First Time: a national review of adult elective orthopaedic services in England*. London: British Orthopaedic Association; 2015.

3. Resar R, Pronovost P, Haraden C, Simmonds T, Rainey T, Nolan T. Using a bundle approach to improve ventilator care processes and reduce ventilator-associated pneumonia. *J Qual Patient Saf*. 2005;31(5):243-8.

4. Maggard-Gibbons M. The use of report cards and outcome measurements to improve the safety of surgical care: the American College of Surgeons National Surgical Quality Improvement Program *BMJ Quality & Safety* 2014;**23:**589-599.

5. Peden CJ, Moonesinghe SR. Measurement for improvement in anaesthesia and intensive care. *Br J Anaesth*. 2016;117(2):145-148.

6. Khan T, Alderson SA, Francis JJ, Lorencatto F, Grant-Casey J, et al. Repeated analyses of national clinical audit reports demonstrate improvements in feedback methods. Implementation Science Communications 2020 ; 1 (1) :1-6

7. Berwick DM. Measuring Surgical Outcomes for Improvement: Was Codman Wrong? *JAMA.* 2015;313(5):469–470. doi:10.1001/jama.2015.4

8. Dixon-Woods M, Bosk CL, Aveling EL, Goeschel CA, Pronovost PJ. Explaining Michigan: developing an ex post theory of a quality improvement program. Milbank Q. 2011;89:167–205.

9. Dixon-Woods M, McNicol S, Martin G. Ten challenges in improving quality in healthcare: lessons from the Health Foundation's programme evaluations and relevant literature. *BMJ Quality & Safety* 2012;**21:**876-884.

10. Cohen ME, Liu Y, Ko CY, Hall BL. Improved Surgical Outcomes for ACS NSQIP Hospitals Over Time: Evaluation of Hospital Cohorts With up to 8 Years of Participation. Ann Surg. 2016 Feb;263(2):267-73. doi: 10.1097/SLA.0000000000001192. PMID: 25723845.

11. Montroy J, Breau RH, Cnossen S, Witiuk K, Binette A, Ferrier T, Lavallée LT, Fergusson DA, Schramm D. Change in Adverse Events After Enrollment in the National Surgical Quality Improvement Program: A Systematic Review and Meta-Analysis. PLoS One. 2016 Jan 26;11(1):e0146254. doi: 10.1371/journal.pone.0146254. PMID: 26812596; PMCID: PMC4727780.

12. Turner S, Ramsay A, Perry C, Boaden R, McKevitt C, Morris S, et al. Lessons for major system change: centralization of stroke services in two metropolitan areas of England. *J Health Serv Res Policy*. 2016;21:156–65.

13. Barratt H, Turner S, Hutchings A, Pizzo E, Hudson E, Briggs T, et al. Mixed methods evaluation of the Getting it Right First Time programme – improvements to NHS orthopaedic care in England: study protocol. *BMC Health Serv Res* 2017;17:71

For peer review only - http://bmjopen.bmj.com/site/about/guidelines.xhtml

14. NHS Digital. Patient Reported Outcome Measure https://digital.nhs.uk/data-and-information/data-tools-and-services/data-services/patient-reported-outcome-measures-proms (Accessed 8 Jun 2021)

15. Matharu G S, Judge A, Pandit H G, Murray D W. Follow-up for patients with metal-on-metal hip replacements: are the new MHRA recommendations justified? *BMJ* 2018; 360 :k566 doi:10.1136/bmj.k566

16. EuroQol Research Foundation. EQ-5D-3L User Guide, 2018. Available from: https://euroqol.org/publications/user-guides (Accessed 8 Jun 2021)

17. Dawson J, Fitzpatrick R, Carr A, Murray D. Questionnaire on the perceptions of patients about total hip replacement. J Bone Joint Surg Br. 1996 Mar;78(2):185-90.

18. Sibanda N, Copley LP, Lewsey JD, et al. Revision rates after primary hip and knee replacement in England between 2003 and 2006, *PLoS Med*, 2008;5:e179

19. Armitage JN van der Meulen JH . Identifying co-morbidity in surgical patients using administrative data with the Royal College of Surgeons Charlson Score. *Br J Surg* 2010; 97 : 772 – 81

20. GIRFT. *Getting It Right in Orthopaedics. Reflecting on success and reinforing improvement: a follow-up on the GIRFT national specialty report on orthopaedics.* London: Getting it Right First Time, 2020. https://gettingitrightfirsttime.co.uk/wp-content/uploads/2020/02/GIRFT-orthopaedics-follow-up-report-February-2020.pdf (Accessed 29 April 2021)

21. NHS Improvement. NHS 2018/19 National Tariff: currencies and prices. https://improvement.nhs.uk/resources/national-tariff (Accessed 8 Jun 2021)

22. Yin RK. Validity and generalization in future case study evaluations. *Evaluation*. 2013;19:321–32

23. Bradley EH, Curry LA, Devers KJ. Qualitative data analysis for health services research: developing taxonomy, themes, and theory. *Health Serv Res*. 2007; 42:1758–72.

24. https://resolution.nhs.uk/wp-content/uploads/2017/08/UCL-final-evaluation-report-bid-scheme.pdf (Accessed 8 Jun 2021)

25. Sedgwick P. What is recall bias? *BMJ* 2012; 344 :e3519 doi:10.1136/bmj.e351

26. Timmins N. *Tackling variations in clinical care: Assessing the Getting It Right First Time (GIRFT) programme*. London: Kings Fund, 2017 https://www.kingsfund.org.uk/sites/default/files/field/field_publication_file/Getting_it_right_Kings_Fund_June_2017.pdf (Accessed 29 April 2021)

27. NHS Providers. *The Getting it Right First Time programme: Early views from the provider sector.* London: NHS Providers, 2018. https://nhsproviders.org/media/4365/girft-final-pdf.pdf (Accessed 29 April 2021)

28. Ivers N, Jamtvedt G, Flottorp S, Young JM, Odgaard-Jensen J, French SD, O'Brien MA, Johansen M, Grimshaw J, Oxman AD. Audit and feedback: effects on professional practice and healthcare

For peer review only - http://bmjopen.bmj.com/site/about/guidelines.xhtml

outcomes. Cochrane Database Syst Rev. 2012 Jun 13;(6):CD000259. doi: 10.1002/14651858.CD000259.pub3. PMID: 22696318.

29. Bion J, Richardson A, Hibbert P THE MATCHING MICHIGAN COLLABORATION & WRITING COMMITTEE*, et al* 'Matching Michigan': a 2-year stepped interventional programme to minimise central venous catheter-blood stream infections in intensive care units in England *BMJ Quality & Safety* 2013;**22:**110-123.

For peer review only

	What	How	Data source
**Cost of GIRFT visits**	Cost of GIRFT team<br>Data pack production<br>Cost of visits trips<br>Cost of visits time<br>Cost of Trusts staff time	Staff wages<br>Cost of data pack<br>Mileage cost x total miles<br>Transport/meeting time x wage/hr<br>Meetings time x wage/hr	NHS, PSSRU<br>GIRFT team data
**Cost for Trusts to implement recommendations**	Ring fenced<br>Extended physiotherapy<br>Theatre load kits<br>Minimum Volumes<br>Theatre Efficiency	National Survey to NHS Trusts	National Survey to NHS Trusts in August 2018
**Economic impact of changes in clinical practice and outcomes**	Arthroscopy procedures<br>Fixation methods<br>Readmissions<br>Revisions<br>Length of Stay<br>PROMS-QALYs	Quantitative analysis results<br>Volumes x costs of procedures	NJR and HES<br>National Tariffs<br>NICE reports<br>HRGs

Figure 1: The economic components of GIRFT orthopaedic evaluation

209x296mm (500 x 500 DPI)

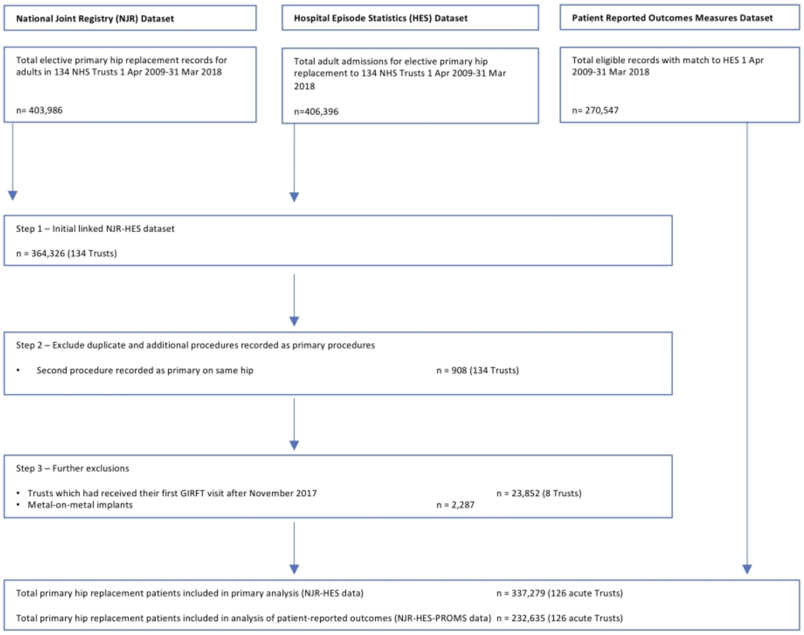

Figure 2: Study population – total hip replacement patients

190x254mm (500 x 500 DPI)

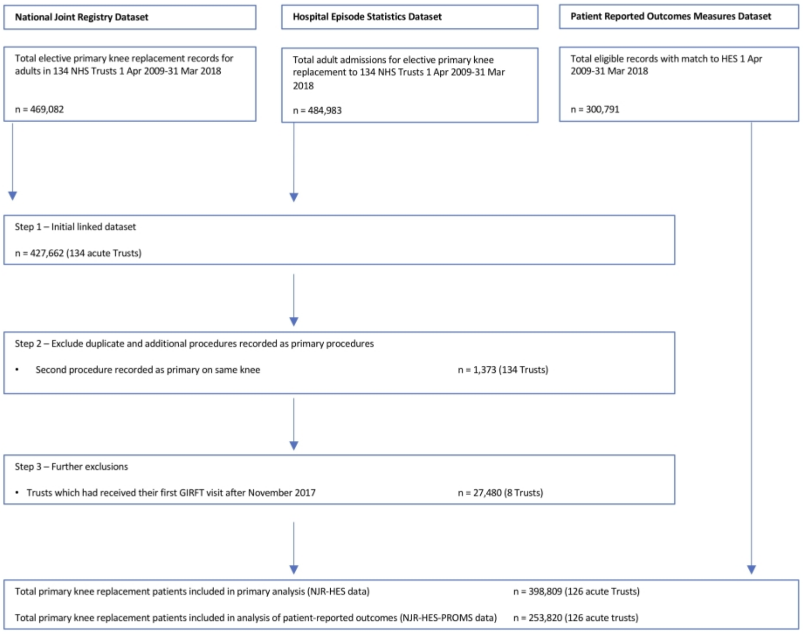

Figure 3: Study population – total knee replacement patients

190x275mm (500 x 500 DPI)

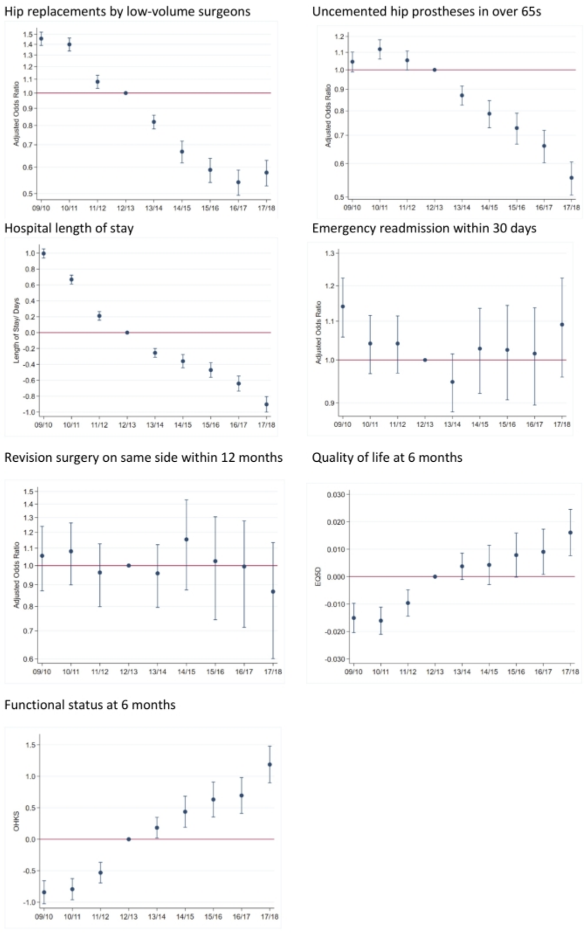

Odds ratios and mean differences reported with 95% confidence intervals from casemix-adjusted hierarchical logistic and linear regression models

Figure 4: Trends in process and outcome measures for primary hip replacements (2009/10-2017/18)

209x296mm (500 x 500 DPI)

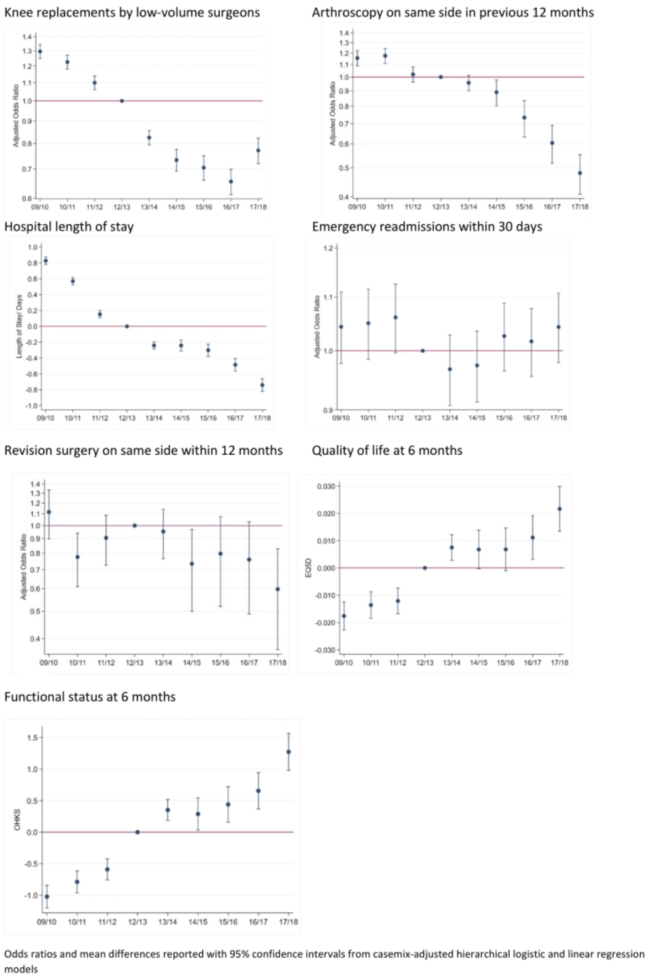

Figure 5: Trends in process and outcome measures for primary knee replacements (2009/10-2017/18)

209x296mm (500 x 500 DPI)

Level of impact	Type of impact	Examples
**Individual practitioner**	1. Changing clinical practice	Reduction in procedures conducted by low volume surgeons (Site 2)<br>Informed choices about implants (e.g. uncemented hips) (Sites 1+3)
**Trust**	2. Catalysing already planned changes	Expedited existing plans to improve implant selection and rationalisation of procurement (Site 3)
	3. Internal data review	Greater use of local data to inform decision-making (All sites)
	4. Influencing ongoing strategic conversations	Helped maintain focus on existing improvement agendas (e.g. ring fenced beds) (All)
	5. Increasing collaborative working	Local: introduction of weekly, multidisciplinary team review of cases (Site 3)
**Regional**		Regional: more networking with neighbouring Trusts (Sites 1+2)

Figure 6: Summary of impacts attributed to GIRFT orthopaedic visits at six case-study site

209x296mm (500 x 500 DPI)

Appendix 1: Case study interview participants

Table A1: Case study interview participants

Site	Orthopaedic surgeons	Nursing staff	Managers	External (e.g. CCG)	Total
1	3	2	3	3	11
2	2	0	7	0	9
3	5	1	1	0	7
4	5*	0	3	0	8
5	6	0	1	0	7
6	3	0	5	0	8
Total	24	3	20	3	50

*Included one spinal surgeon

For peer review only

Appendix 2: Case study topic guide

Topic guide development:

The topic guide for the provider interviews was initially developed through preliminary discussions with the GIRFT programme team (to understand the programme, its programme theory, and any perceived facilitators or barriers to implementation) and the wider research team (to ensure that questions about resource costs and implementation were incorporate to facilate the quantitative and economic analyses).

The draft provider topic guide was finalised in January 2017 and piloted at one of the case study sites. It worked well and was consequently used across all sites. The topic guide was then iteratively refined during the course of the study, in response to both our preliminary findings and the evolution of the GIRFT programme as it was rolled out across the country.

Summary topic guide for provider interviews:

- Awareness of GIRFT programme in orthopaedics
  - First aware of GIRFT?

- Involvement in a GIRFT visit to this site?

- First visit by the GIRFT team
  - Experiences - When, duration, preparation, views on datapacks, main issues raised - helpful/challenging?, expectations, differences to other programmes
  - Actions following first visit

- GIRFT re-visit
  - Prompts as above plus – differences from first visit

- Other local/national quality improvement initiatives
  - Level of involvement, adoption, impact; Model Hospital

- Impact of GIRFT
  - Organisational practice, timescales for changes to practice, changes to resource allocation, help from GIRFT team to deliver changes
  - Attribution to GIRFT
  - Unintended consequences

- Monitoring and measuring change – cost savings, NJR compliance, less low volume procedures, infection rates.

- Other local contextual information
  - Local coding, specific local difficulties in implementing GIRFT recommendations

- Suggestions for improvements to GIRFT programme

- Implementation of GIRFT recommendations since 1st visit (in relation to key metrics)
  - How facilitated, duration, barriers/facilitators
  - Any GIRFT recommendation particularly difficult to implement locally
  - Any local controversies about particular GIRFT recommendations locally
  - Impact of these

For peer review only

- Monitoring and measuring progress against GIRFT recommendations
- Attribution to GIRFT

- Awareness of recent changes to GIRFT programme
  - Involvement with implementation hubs; GIRFT national/regional involvement

For peer review only

STROBE Statement—Checklist of items that should be included in reports of ***cohort studies***

	Item No	Recommendation
**Title and abstract**	1	(*a*) Indicate the study's design with a commonly used term in the title or the abstract<br>**We describe the study as a mixed methods evaluation to indicate the study's overall design (title)**
		(*b*) Provide in the abstract an informative and balanced summary of what was done and what was found<br>**The abstract provides an overview of our methods and results.**
**Introduction**		
Background/rationale	2	Explain the scientific background and rationale for the investigation being reported<br>**We describe GIRFT (the 'intervention'), its context and the justification for the evaluation in the Introduction paragraphs 1-4.**
Objectives	3	State specific objectives, including any prespecified hypotheses<br>**We describe the objectives of the evaluation in the final paragraph of the Introduction.**
**Methods**		
Study design	4	Present key elements of study design early in the paper<br>**We include a summary of the overall design in the first (Design) paragraph of the Methods.**
Setting	5	Describe the setting, locations, and relevant dates, including periods of recruitment, exposure, follow-up, and data collection<br>**We describe the setting/locations/dates for the quantitative analysis in Methods (Quantitative methods: Data sources) and the case study sites (Qualitative methods: Case study sites and Data collection sections). Additional contextual information is provided in the Introduction.**
Participants	6	(*a*) Give the eligibility criteria, and the sources and methods of selection of participants. Describe methods of follow-up<br>**We report eligibility of NHS Trusts and patients for the quantitative analysis in Quantitative methods: Data sources and include information on the pre- and post-intervention data from Trusts. We provide a reference to details of selection of interviewees in Qualitative methods: data collection.**
		(*b*) For matched studies, give matching criteria and number of exposed and unexposed<br>**Not applicable**
Variables	7	Clearly define all outcomes, exposures, predictors, potential confounders, and effect modifiers. Give diagnostic criteria, if applicable<br>We report outcomes and potential confounders in Quantitative methods: Variables.<br>**We describe the 'exposure' as the period beginning with a Trust's first GIRFT visit in Quantitative methods: Statistical analysis as well as the potential modifying effect of the timing of the visit.**
Data sources/ measurement	8*	For each variable of interest, give sources of data and details of methods of assessment (measurement). Describe comparability of assessment methods if there is more than one group<br>**We report the routine data sources and definitions used in Quantitative methods: Data sources and Variables, which is the same for 'exposed' (post-) and 'unexposed' (pre-) periods in individual Trusts.**
Bias	9	Describe any efforts to address potential sources of bias<br>**We describe how we sought to control for temporal trends, non-random timing**

For peer review only - http://bmjopen.bmj.com/site/about/guidelines.xhtml

		**of GIRFT visits and 'washout' periods in Quantitative methods: Statistical analysis.**
Study size	10	Explain how the study size was arrived at<br>**The size of study was determined by availability of routine data rather than recruitment to a target sample size.**
Quantitative variables	11	Explain how quantitative variables were handled in the analyses. If applicable, describe which groupings were chosen and why<br>**We describe how independent variables were handled in Quantitative methods: Variables and how the Trusts were grouped by visit date in Quantitative methods: Statistical analysis.**
Statistical methods	12	(*a*) Describe all statistical methods, including those used to control for confounding<br>**We describe the statistical methods, including those used to control for confounding, in Quantitative methods: Statistical analysis.**
		(*b*) Describe any methods used to examine subgroups and interactions<br>**We describe the statistical methods, including those used to compare the different groups of NHS Trusts, in Quantitative methods: Statistical analysis.**
		(*c*) Explain how missing data were addressed<br>**We dealt with missing data using a complete case analysis. The exception was ethnicity where the missing/not reported category was included in the analysis. We report this in Quantitative methods: Variables and Statistical analysis and missing data are reported in Table 1.**
		(*d*) If applicable, explain how loss to follow-up was addressed<br>**Not applicable**
		(*e*) Describe any sensitivity analyses<br>**Not applicable**
**Results**		
Participants	13*	(a) Report numbers of individuals at each stage of study—eg numbers potentially eligible, examined for eligibility, confirmed eligible, included in the study, completing follow-up, and analysed
		(b) Give reasons for non-participation at each stage
		(c) Consider use of a flow diagram<br>**We have included flow diagrams (Figures 2+3), which set out the number of individuals included at each stage of the study (a), including reasons for non-participation (b).**
Descriptive data	14*	(a) Give characteristics of study participants (eg demographic, clinical, social) and information on exposures and potential confounders<br>**Table 1 describes the characteristics of study participants, including potential confounders.**
		(b) Indicate number of participants with missing data for each variable of interest<br>**This information is provided in Table 1.**
		(c) Summarise follow-up time (eg, average and total amount)<br>**Not applicable.**
Outcome data	15*	Report numbers of outcome events or summary measures over time<br>**We report summary measures of the outcome events over time in Table 2 and Figures 4+5**
Main results	16	(*a*) Give unadjusted estimates and, if applicable, confounder-adjusted estimates and their precision (eg, 95% confidence interval). Make clear which confounders were adjusted for and why they were included
		(*b*) Report category boundaries when continuous variables were categorized

For peer review only - http://bmjopen.bmj.com/site/about/guidelines.xhtml

		**This information is reported in Quantitative methods: variables, and also in Table 1.**
		(*c*) If relevant, consider translating estimates of relative risk into absolute risk for a meaningful time period<br>**Not applicable.**
Other analyses	17	Report other analyses done—eg analyses of subgroups and interactions, and sensitivity analyses<br>**Not applicable.**
**Discussion**		
Key results	18	Summarise key results with reference to study objectives<br>**Key results are summarised in the first paragraph of the Discussion.**
Limitations	19	Discuss limitations of the study, taking into account sources of potential bias or imprecision. Discuss both direction and magnitude of any potential bias<br>**Limitations of the study, including potential sources of bias, are listed in paragraph 2 of the Discussion.**
Interpretation	20	Give a cautious overall interpretation of results considering objectives, limitations, multiplicity of analyses, results from similar studies, and other relevant evidence<br>**We provide an interpretation of the results in the penultimate paragraph of the Discussion. We set the findings in the context of other research in the third paragraph of the Discussion.**
Generalisability	21	Discuss the generalisability (external validity) of the study results<br>**We discuss the generalisability of the results to other improvement initiatives in paragraphs 4+5 of the Discussion.**
**Other information**		
Funding	22	Give the source of funding and the role of the funders for the present study and, if applicable, for the original study on which the present article is based<br>**We have included statements about the source of funding and the role of the funder in the author statements at the end of the article.**

*Give information separately for exposed and unexposed groups.

Note: An Explanation and Elaboration article discusses each checklist item and gives methodological background and published examples of transparent reporting. The STROBE checklist is best used in conjunction with this article (freely available on the Web sites of PLoS Medicine at http://www.plosmedicine.org/, Annals of Internal Medicine at http://www.annals.org/, and Epidemiology at http://www.epidem.com/). Information on the STROBE Initiative is available at http://www.strobe-statement.org.

For peer review only
